# Adjacent mutations in the archaeal Rad50 ABC ATPase D-loop disrupt allosteric regulation of ATP hydrolysis through different mechanisms

**DOI:** 10.1093/nar/gkz1228

**Published:** 2019-12-31

**Authors:** Zachary K Boswell, Marella D Canny, Tanner A Buschmann, Julie Sang, Michael P Latham

**Affiliations:** Department of Chemistry and Biochemistry, Texas Tech University, Lubbock, TX 79409-1061, USA

## Abstract

DNA damage is the driving force for mutation and genomic instability, which can both lead to cell death or carcinogenesis. DNA double strand breaks are detected and processed in part by the Mre11–Rad50–Nbs1 protein complex. Although the Mre11–Rad50–Nbs1 complex is essential, several spontaneous mutations have been noted in various cancers. One of these mutations, within a conserved motif of Rad50, resulted in an outlier curative response in a clinical trial. We show through biochemical and biophysical characterization that this cancer-associated mutation and a second mutation to the adjacent residue, previously described in a breast cancer patient, both have gain-of-function Rad50 ATP hydrolysis activity that results not from faster association of the ATP-bound form but faster dissociation leading to less stable Rad50 dimer. This disruption impairs the regulatory functions of the protein complex leading to a loss of exonuclease activity from Mre11. Interestingly, these two mutations affect Rad50 structure and dynamics quite differently. These studies describe the relationship between function, structure, and molecular motions in improperly regulated Rad50, which reveal the underlying biophysical mechanism for how these two cancer-associated mutations affect the cell.

## INTRODUCTION

The integrity and stability of a genome is under constant assault from a number of internal and external stresses, such as reactive oxygen species, replicative stress, UV light, and genotoxic chemicals. Failure to detect and accurately repair the various DNA lesions that result from these assaults can lead to simple mutations and/or large scale chromosomal rearrangements, which could drive the development of cancer in humans. Fortunately, the cell has developed sophisticated systems to find and repair each distinct form of DNA damage. DNA double strand breaks, where both strands of the DNA double helix are broken, are a particularly dangerous form of DNA damage, as a template for accurate repair is not readily available. Thus, the detection and repair of DNA double strand breaks are critical for cellular survival and cancer prevention (1).

**Figure 1.**
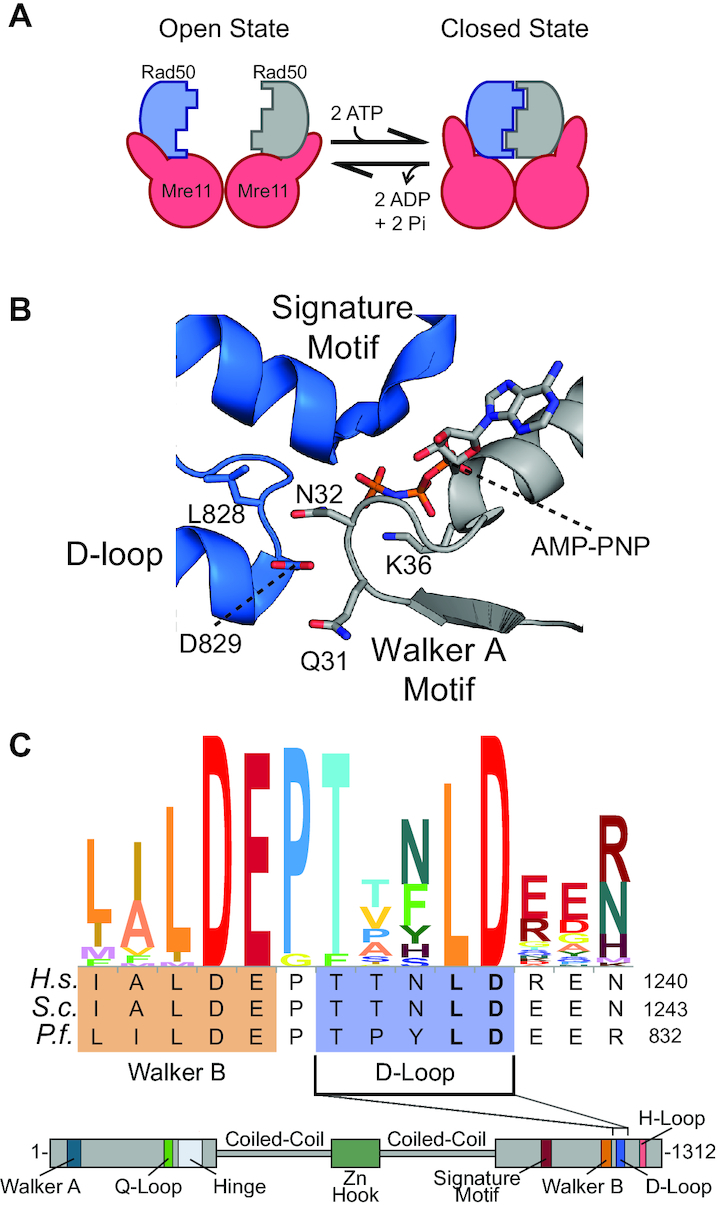
The Rad50 D-loop. (**A**) Cartoon representation of the ATP-induced global structural change of the MR complex. The Mre11 dimer is red, and the two protomers of Rad50 are blue and gray. (**B**) Structure of the ATP-bound closed form of *P. furiosus* Rad50 NBD (pdb: 3QKU (15)) focusing on the D-loop (blue protomer)–Walker A (gray protomer) interaction. (**C**) An alignment of 32 Rad50 sequences, focusing on the Walker B and D-loop motifs, produced with Skylign (55) (top) and a cartoon representation of the conserved ABC ATPase motifs in Rad50 (bottom). *H. sapiens*, *S. cerevisiae* and *P. furiosus* Rad50 sequences are given below the Skylign representation.

Mre11–Rad50–Nbs1 (MRN) is an essential protein complex that is a primary responder to DNA double strand breaks, hairpins, and other anomalous terminal DNA structures. MRN participates in tethering damaged DNA strands and preparing the broken ends for repair by downstream homologous recombination or non-homologous end joining pathways (2,3). The MRN complex utilizes a variety of functions in the process of detecting and initiating DNA double strand break repair. Mre11 functions as a dimer and has Mn^2+^-dependent exo- and endonuclease activities (4–6). The endonuclease activity in particular is important for the removal of DNA-protein adducts that are formed from failed topoisomerase reactions (7). One Rad50, a member of the ATP-Binding Cassette (ABC) ATPase super-family, binds to each of the Mre11 protomers and regulates the conformational and functional states of MRN via ATP-induced association of the two Rad50 nucleotide binding domains (NBDs) and subsequent ATP hydrolysis (2,3,8). The eukaryotic Nbs1 (or Xrs1 in budding yeast) is a signaling hub associated with Mre11 that recruits downstream effectors to the site of damage (9–11). In the universally conserved Mre11_2_–Rad50_2_ (MR) core complex, Rad50 undergoes dramatic conformational changes in response to ATP binding and hydrolysis (12–15). In the ATP-free ‘open’ conformation, Mre11 is an active nuclease (Figure 1A; left); whereas, in the ATP-bound ‘closed’ conformation (Figure 1A; right), Mre11 active sites are occluded. Additionally, Rad50 binds DNA in the ATP-bound ‘closed’ conformation, a function that is important for telomere maintenance (16–18). Rad50 ATP hydrolysis returns the complex to the ‘open’ conformation and leads to increased and processive exonuclease activity and endonuclease activity to remove DNA–protein adducts (7,19,20). Interestingly, the presence of DNA accelerates the rate of Rad50 ATP hydrolysis *in vitro* (21,22).

A vast allosteric network within Rad50 governs the rate of ATP hydrolysis and therefore the ATP-dependent global conformational changes (Figure 1A) and activity of the MR complex (15,18,23,24). This network spans a DNA-binding site, the hinge and ‘basic switch,’ the Q-loop, the Walker B motif, and the ABC signature motif within one protomer. In the ATP-bound ‘closed’ conformation, this network then extends to the other protomer via an interaction between the D-loop and *trans*-Walker A motif (Figure 1B), which is mediated through hydrogen bonds between the side chain of one protomer and the backbone of the other. For example, in the crystal structure of the ‘closed’ conformation of *P. furiosus* Rad50 NBD (PDB ID: 3QKT; Figure 1B), the conserved aspartate (D829) forms a backbone hydrogen bond with the amide group of Walker A residue N32; whereas, the Walker A residues Q31 and N32 form backbone hydrogen bonds with D829 and E830. In addition to the D-loop–Walker A interaction, the two Rad50 NBDs in the complex are also held in the ATP-bound ‘closed’ conformation by interactions between the ATP γ-phosphate and the *trans*-Rad50 ABC signature motif (8,25). Disruption of this network via mutation of residues in the hinge and ‘basic switch’ region leads to increased Rad50 ATP hydrolysis and Mre11 exonuclease activities, underscoring the importance of this network in MR function (18,23). Within this allosteric network, the precise role of the universally conserved D-loop motif (Figure 1B) remains unclear. Mutations of the adjacent and conserved Rad50 D-loop leucine and aspartate residues have been found in solid metastatic and breast cancer cells, respectively (26). Significantly, a leucine to phenylalanine mutation resulted in a curative outlier response for a patient in a clinical trial using a combination of a topoisomerase I inhibitor and a cell cycle checkpoint (CHK1/2) inhibitor. Intriguingly, the characterization of these mutations in *S. cerevisiae* and mouse embryonic fibroblast cells revealed that both of these D-loop mutations lead to a loss of ATM kinase activation, disrupting DNA double strand break repair (26).

To investigate the biochemical and structural underpinning for the altered functions of these mutations, we used *Pyrococcus furiosus* (*Pf*) MR as a model system to characterize *Pf* Rad50 L828F and D829N mutations. *Pf* Rad50 is highly conserved in the critical ABC motifs (Figure 1C), and previous studies have proven transitive to eukaryotic systems (5,6,15,18). Moreover, the thermostability of the enzyme allowed us to perform nuclear magnetic resonance (NMR) experiments at elevated temperatures which improves the signal-to-noise and resolution of data collected on the ∼180 kDa MR complex. We also investigated the effects of non-disease related alanine substitutions at these two D-loop positions. For this study, we employed the full-length *Pf* MR complex for biochemical assays and a commonly used truncated form of *Pf* Rad50, lacking the majority of the coiled-coil and the Zn-hook domains (Rad50^NBD^; Figure 1C, lower) for NMR studies (15,23). Here, we show that D-loop mutants have gain-of-function ATP hydrolysis activity, while paradoxically, forming less of the ATP-induced ‘closed’ conformation—the state that is necessary for ATP hydrolysis. Using methyl-based solution-state NMR spectroscopy, we characterized the effect of each mutation on the structure and fast timescale dynamics of Rad50 NBD. The L828F and L828A mutations affected the structure and dynamics of methyl groups throughout Rad50; whereas, the D829N mutation only affected the chemical environment and dynamics of methyl groups in the immediate vicinity of the mutation. In contrast, the D829A mutation showed the largest changes to the structure and dynamics of the NBD. Furthermore, we demonstrated that the D-loop mutants altered the nuclease activity of the MR complex. Based on our data, we propose a model for the allosteric regulation of Rad50 ATP hydrolysis that describes how each of these mutations disrupt ATM signaling in cancer cells. Finally, our data suggests a strategy for the design of novel small molecules that can modulate the activity of Rad50 and interrupt ATM signaling, possibly providing another approach for synthetic lethality in anti-cancer therapies.

## MATERIALS AND METHODS

### Protein expression, purification, and Mre11_2_–Rad50_2_ (MR) complex formation

Unlabeled and U-^2^H, Ileδ1-[^13^CH_3_], Leuδ/Valγ-[^13^CH_3_,^12^CD_3_], Metϵ-[^13^CH_3_] (ILVM)-labeled (27) *P. furiosus* (*Pf*) Rad50 nucleotide binding domain (Rad50^NBD^; amino acids 1–195—GGAGGAGG [linker]—709–882) was expressed and purified as previously described (23,28). The gene for full-length *Pf* Rad50 was cloned out of *P. furiosus* genomic DNA (ATCC) and inserted into a modified pET-29 vector (Novagen) with an N-terminal TEV protease-cleavable 6x-His tag. Full-length Rad50 was expressed overnight at 18°C in Rosetta (DE3) competent cells (Novagen). Cells were lysed via sonication in 25 mM HEPES, 300 mM KCl, 25 mM imidazole, 10% glycerol, 20 mM βME, pH 7 buffer and after heat denaturation and centrifugation to pellet the denatured proteins, the full-length Rad50 protein was purified on a HisTrap (GE Healthcare) column and eluted in 300 mM imidazole. After TEV-cleavage of the 6xHis tag, full-length Rad50 was re-purified over the HisTrap column. At this point, the sample was diluted to bring the concentration of KCl down to 75 mM, was loaded onto a HiTrapQ HP (GE Healthcare) column, and eluted in a linear gradient into 25 mM Tris, 1 M NaCl, 10% glycerol, 1 mM DTT, pH 8. 0.2% Tween-20 was added to the pooled Q peak fractions before concentrating (Vivaspin, Sartorius) the protein down to 1 ml to load on a Superdex 200 HILoad 16/600 (GE Healthcare) gel filtration column equilibrated in 25 mM Tris, 100 mM NaCl, 10% glycerol, 1 mM TCEP, pH 8. Point mutations to Rad50 were generated via a modified Stratagene Quikchange protocol and were verified via Sanger sequencing. All L51C mutant purification buffers contained either 10 mM β-mercaptoethanol or 1 mM TCEP (tris(2-carboxyethyl)phosphine) to keep the cysteine reduced.

The *Pf* Mre11 gene, codon optimized for expression in *Escherichia coli*, was synthesized by Life Technologies and expressed from a modified pET-29 vector (Novagen) with an N-terminal TEV protease-cleavable 6x-His tag in *E. coli* BL21 (DE3) C41 cells (Sigma). Mre11 protein was purified to homogeneity by heat denaturation, HisTrap (GE Healthcare) nickel affinity, HiTrapQ HP (GE Healthcare) anion exchange, and Superdex 200 HiLoad 16/600 (GE Healthcare) gel filtration chromatography. Mre11^HLH^ was purified as described (23).

MR complexes for activity and binding assays were formed by mixing purified Mre11 and full-length Rad50 or Rad50^NBD^ in a 2:2 molar ratio and heating at 65°C for 10 min before cooling to room temperature.

### ATP hydrolysis

The steady-state kinetics of *Pf* MR complex ATP hydrolysis were measured via the BIOMOL Green (Enzo Lifesciences) colorimetric assay as previously described (23) with some modifications. 60 μl reactions containing either full-length MR or truncated MR^NBD^ and 0–300 μM ATP were incubated for 60 min at 65°C. For full-length MR, hydrolysis reactions contained 2.5 μM MR complexes in the absence of DNA or 1.25 μM MR complexes in the presence of DNA. For truncated MR^NBD^, 2.5 μM wild type, 1.25 μM L828F, 625 nM D829N, 2.5 μM L828A or 1.25 μM D829A were present for the hydrolysis reactions in the absence of DNA, and 2.5 μM wild type, 1.25 μM L828F, 625 nM D829N, 1.25 μM L828A or 0.625 μM D829A were used in the presence of DNA. Buffer contained 50 mM Tris, 80 mM NaCl, 1% glycerol and 5 mM MgCl_2_, pH 7.5. Reactions with DNA contained 25 nM of ∼5 kb DNA (pRS416, Addgene) that was linearized with EcoR1 (New England BioLabs), ethanol precipitated, and resuspended in water. The phosphate concentration-dependent absorbance of the BIOMOL reagent for each condition (i.e., no protein blank, with MR, and with MR and DNA) was converted to μM PO_4_ μM MR^−1^ h^−1^ via individual standard curves. Initial velocities (*v*) were fit to the Hill equation

$$\begin{equation*}v\ = \ \frac{{{V_{{\rm max}}}{{[ {{\rm ATP}} ]}^n}}}{{K_{\rm M}^n + \ {{[ {{\rm ATP}}]}^n}}}\end{equation*}$$

where *V*_max_ is the maximal velocity, [ATP] is the concentration of ATP, *K*_M_ is the Michaelis constant and *n* is the Hill coefficient. Plots are the average of three replicate experiments using standard deviations as the reported errors.

### LRET

Purified *Pf* Rad50^NBD^ L51C and L51C/D-loop mutant proteins were mixed with a 2-fold molar excess of either the thiol-reactive Tb^3+^ chelate DTPA-cs124-EMPH (donor, Lanthascreen, Life Technologies, Inc.) or Bodipy FL maleimide (acceptor, Invitrogen) in the presence of 1 mM TCEP. Labeling reactions were incubated at room temperature for 1–2 h in the dark. Excess label was removed via size exclusion chromatography on a Superdex 200 Increase 10/300 GL column (GE Healthcare) equilibrated with 25 mM HEPES, 200 mM NaCl, 0.1 mM EDTA, pH 7.0. LRET labeling of full-length *Pf* Rad50 L51C and L51C/D-loop mutants was essentially the same, except the Tb^3+^ chelate and Bodipy FL labels were added together at the same time and the S200 column to remove excess label was equilibrated in 50 mM HEPES, 100 mM NaCl, 10% glycerol, 1 mM TCEP, pH 7.5.

LRET emission spectra in the absence or presence of 2 mM ATP were recorded with a Photon Technology International spectrometer (QM3SS) using a 3-mm pathlength quartz cuvette, with 0.5 μM wild type, L828A, and D829A MR^NBD^ complexes (i.e. 0.5 μM Tb^3+^-labeled Rad50^NBD^, 0.5 μM Bodipy FL-labeled Rad50^NBD^ and 1.0 μM Mre11) or 2.0 μM L828F and D829N MR^NBD^ complexes, in 150 μl of 50 mM Tris, 200 mM NaCl, 5 mM MgCl_2_, pH 7.5 at 50°C. LRET experiments on full-length Rad50 constructs all contained 0.5 μM MR complex where dual-labeled full-length Rad50 was mixed 1:1 with Mre11. The luminescent Tb^3+^ chelate was excited with a ∼1-μs pulse from a Xenon flash lamp at 337 nm, and emission scans were measured from 470–570 nm after a 200 μs delay. The spectra were normalized to the Tb^3+^ emission peak at 549 nm as its emission remains constant in this experiment. The signal at ∼515 nm corresponds to donor-sensitized Bodipy FL emission.

Tb^3+^ and Bodipy FL lifetimes were calculated from intensity decays measured with an Optical Building Blocks phosphorescence lifetime photometer (EasyLife L) in the same conditions as above. After excitation at 335 nm through a narrow band filter (Semrock FF01-335/7) and a 200 μs delay, donor emission intensity was collected for Tb^3+^ at 50 Hz through a 490/10 nm band-pass filter (Omega Optical) while donor-sensitized acceptor emission of Bodipy FL was collected at 100 Hz through a 520/10 nm band-pass filter (Omega Optical). Bodipy FL emission decay curves were used to calculate donor-sensitized lifetime distributions using the exponential series method (ESM) (29) in the PTI FeliX32 software package. To derive the distributions, the ESM method used 200 logarithmically-spaced lifetime bins ranging from 100 to 1500 μs. Bodipy FL emission decay curves were also fit to a three-exponential function in PTI FeliX32. In both these analyses, lifetime distributions shorter of ∼100 μs were discarded as these are largely a function of the instrument response time (30–32). The distances between donor and acceptor molecules (*R*) were subsequently calculated from these lifetimes with

$$\begin{equation*}E\ = \ 1 - {\raise0.7ex\hbox{${{\tau _{{\rm DA}}}}$} \!\mathord{\left/ {\vphantom {{{\tau _{DA}}} {{\tau _{\rm D}}}}}\right.} \!\lower0.7ex\hbox{${{\tau _D}}$}}\end{equation*}$$

and

$$\begin{equation*}R\ = {R_0}\ \ {\left( {{E^{ - 1}} - 1} \right)^{1/6}}\end{equation*}$$

where *E* is the efficiency of energy transfer, *τ*_DA_ is the donor-sensitized lifetime of the acceptor (i.e. Bodipy FL), *τ*_D_is the lifetime of the donor (i.e. Tb^3+^), and *R*_0_ is the Förster distance between Tb^3+^ and Bodipy FL (44.9 Å). Errors are the standard deviations of at least three measurements.

The equilibration rate constant (*k*_ex_ = *k*_assoc_ + *k*_dissoc_) for the approach of the ATP-induced MR complex ${\rm open}\ {\rightleftharpoons\!\!\!\!\!\!\!\!}^{^{^{^{^{k_{\rm assoc}}}}}}_{_{_{_{_{k_{\rm dissoc}}}}}}\ {\rm closed}$ to equilibrium was calculated from the increase in donor-sensitized Bodipy FL emission over time after the addition of 2 mM ATP in the same conditions as described above. Bodipy FL emission was continuously recorded, as described above, until the signal fully plateaued: ∼10, ∼3, ∼2 and ∼3 min for wild type, L828F, D829N, L828A MR^NBD^ complexes respectively. *k*_ex_ were calculated from fits of the recorded intensities (*F*) by

$$\begin{equation*}F\ = \left( {{F_{{\rm max}}} - \ {F_0}} \right)\ \left( {1 - \ {{\rm e}^{ - {k_{{\rm ex}}}t}}} \right) + {F_0}\end{equation*}$$

where *F*_max_ is the maximum intensity, *F*_0_ is the initial intensity, and *t* is time. Errors are from the covariance in the fit.

### Chemical shift perturbations

NMR spectra of wild type, L828F, D829N, L828A and D829A *Pf* MR^NBD^ complexes were recorded on U-^2^H, Ileδ1-[^13^CH_3_], Leuδ/Valγ-[^13^CH_3_,^12^CD_3_], Metϵ-[^13^CH_3_] ILVM-labeled Rad50 in complex with unlabeled Mre11 in deuterated 25 mM HEPES, 150 mM ammonium sulfate, 100 mM sodium acetate, pH 7 (uncorrected). 2D ^13^C,^1^H methyl-Transverse Relaxation Optimized Spectroscopy (TROSY) Heteronuclear Multiple Quantum Correlation (HMQC) spectra (33,34) were recorded at 60°C on a 600 MHz (14.1 T) Agilent DD2 NMR spectrometer equipped with a room temperature z-axis gradient probe; data were processed with NMRPipe and analysed with CCPN analysis (35,36).

Chemical shift perturbations (*CSPs*) were determined by calculating distances between the ^13^C (δ_C_) and ^1^H (δ_H_) chemical shifts for wild type (wt) and mutant (mut) protein:

$$\begin{equation*}{\rm CSPs}\ = \ \sqrt {{{\left( {\frac{{{\delta _{{\rm C},{\rm wt}}} - \ {\delta _{{\rm C},{\rm mut}}}}}{{{{ w}_{\rm C}}}}} \right)}^2} + \ {{\left( {\frac{{{\delta _{{\rm H},{\rm wt}}} - \ {\delta _{{\rm H},{\rm mut}}}}}{{{w_{\rm H}}}}} \right)}^2}} \end{equation*}$$

where *w*_C_= (1.65, 1.6, 1.4 and 1.54) and *w*_H_ = (0.29, 0.28, 0.27 and 0.41) are the standard deviations for the side chain methyl group Ileδ1, Leuδ, Valγ and Metϵ chemical shifts (δ_C_ and δ_H_) from the Biological Magnetic Resonance data bank. CSP outliers, which were values above the mean, are reported (i.e. CSPs ≥ 20 ppb; parts per billion). We assume that peaks that do not change position or have small CSPs (< 20 ppb) are not affected from mutation.

### Nano- to picosecond timescale protein dynamics

Side chain methyl group ^1^H ‘forbidden’ Triple Quantum coherence relaxation rates (37) were measured at 60°C on a 600 MHz (14.1 T) Agilent DD2 NMR spectrometer equipped with a room temperature z-axis gradient probe. Relaxation delays (*T*) of 2, 4, 6, 8, 10, 15, 20, 25 and 30 ms were recorded for the ‘allowed’ and ‘forbidden’ transitions in an interleaved manner. Intra-methyl ^1^H–^1^H dipolar cross-correlated relaxation rates (*η*) were calculated by fitting ratios of ‘forbidden’ and ‘allowed’ intensities as a function of *T*,

$$\begin{equation*}\ \left| {\frac{{{I_{{\rm forbid}}}}}{{{I_{{\rm allow}}}}}} \right| = \ \frac{{C\ \eta \tanh\left( {\sqrt {{\eta ^2} + \ {\delta ^2}} T} \right)}}{{\sqrt {{\eta ^2} + \ {\delta ^2}} \ - \ \delta \tanh\left( {\sqrt {{\eta ^2} + \ {\delta ^2}} T} \right)}}\end{equation*}$$

where *I*_forbid_ and *I*_allow_ are the intensities of the ^1^H triple- (‘forbidden’) and single- (‘allowed’) quantum coherences, respectively, *C* = 0.75, and *δ* accounts for the relaxation from external protons. The reported errors were calculated from the covariance of the fit. Changes in dynamics, which were above the mean change, (i.e. |Δ*η*| > 16 s^−1^) are reported.

### Mre11 nuclease activity assays

*Pf* MR complex exonuclease activity was determined using a 29-nucleotide substrate with a 2-aminopurine at the second nucleotide from the 3′-end (Exo2) as previously described (23). MR^NBD^ reactions containing 0.5 μM MR^NBD^ and 1 μM Exo2 dsDNA substrate in 50 mM Tris, 150 mM NaCl, 0.1% PEG-6000 and 2.5% glycerol, pH 7.5 buffer were incubated at 60°C for 45 min in the presence of 1 mM MnCl_2_/5 mM MgCl_2_ or 1 mM MnCl_2_/5 mM MgCl_2_/1 mM ATP or 1 mM MnCl_2_/5 mM MgCl_2_/1 mM AMP-PNP. For full-length MR, the exonuclease assay was essentially the same, except the reaction buffer was 50 mM HEPES, 150 mM NaCl, 0.1% PEG-6000, 2.5% glycerol, 1 mM DTT, pH 7. Reactions contained 0.5 μM full-length MR complex and 1 μM of either the Exo2 dsDNA substrate or the Exo11 dsDNA substrate (5′-GGCGTGCCTTGGGCGCGC[2AmPur]GCGGGCGGAG-3′ annealed to 5′-CTCCGCCCGCTGCGCGCCCAAGGCACGCC-3′) and were incubated at 60°C for 30 min. 2-Aminopurine fluorescence (ex310/em375) was measured with a Synergy Neo2 plate reader. The reported fluorescence values are the average and standard deviation (error bars) of three replicates.

Endonuclease reactions were performed using 1 μg of ΦX174 single stranded virion circular DNA (New England Biolabs) as the substrate in 30 μl reactions. 0.5 μM MR^NBD^ complex in 50 mM Tris, 150 mM NaCl, 2.5% glycerol, 0.1% PEG-6000, 5 mM MgCl_2_, 1 mM DTT, pH 7.5 (without or with 1 mM MnCl_2_ and 1 mM ATP) was incubated at 60°C. For full-length MR, 1 μM complex was incubated in the same conditions, except with 50 mM HEPES pH 7 as the buffer instead of the Tris. 6 μl time points were removed at 5, 10, 20 and 30 min and were stopped by adding 1% (w/v) SDS, 10 mM EDTA, 0.3 mg/ml Proteinase K. Endonuclease products were resolved on 1% agarose gels (in 1× TAE) run at 150 V for 50 min, stained with GelRed nucleic acid stain (Biotium), and imaged with UV light.

### DNA end tethering

The phagemid pRS413 plasmid (Addgene) was linearized with EcoRI (New England Biolabs), to generate 4 nucleotide 5′ overhangs, incubated for 30 min at 60°C, ethanol precipitated, and resuspended in MQ water. 10 μl reactions of 100 ng linearized DNA and 1 μM full-length MR or MR^NBD^ in 50 mM Tris pH, 80 mM NaCl, 5 mM MgCl_2_, 1% glycerol, 2 mM ATP, pH 7.5 were incubated for 10 min at 65°C. Reactions were then cooled to room temperature, and 1 unit of T4 DNA ligase (New England Biolabs) with 1× ligase buffer was added. Ligation reactions proceeded for 30 min at 16°C. Reactions were quenched with 0.2% SDS and 10 mM EDTA and were incubated with 4 μg of Proteinase K at 37°C for one hour. Linear DNA and multimeric tethering products were resolved via 0.5% agarose gel electrophoresis (in 1× TAE) at 150 V for 2 h, stained with Gel Red nucleic acid stain (Biotium), and imaged with UV light.

### ATP binding

*Pf* MR complex ATP binding affinity was measured as previously described (23) with slight modification. Briefly, 5 nM Bodipy FL ATP was titrated with either 0–25 μM MR^NBD^ (i.e. 0–50 μM Rad50 ATP binding sites) or 0–22.5 μM full-length MR. Fluorescence polarization of Bodipy FL ATP (Life Technologies) was measured at room temperature, to limit ATP hydrolysis, in a Synergy Neo2 multi-mode reader (BioTek) using a FP 485/530 filter. Binding affinities were calculated by fitting polarization (*F*) versus protein concentration ([*P*]) to the two-state quadratic binding function,

$$\begin{eqnarray*}F\ &=& \ {F_0} + \left( {{F_{{\rm max}}} - \ {F_0}} \right)\nonumber\\ &&\times\,\frac{{\left( {{K_{\rm D}} + \left[ {{\rm ATP}} \right] + \left[ P \right]} \right) - \ \sqrt {{{\left( {{K_{\rm D}} + \left[ {{\rm ATP}} \right] + \left[ P \right]} \right)}^2} - 4\ \left[ {{\rm ATP}} \right]\left[ P \right]} }}{{2\ \left[ {{\rm ATP}} \right]}}\end{eqnarray*}$$

where *F*_0_ and *F*_max_ are the initial and final polarization values, [ATP] is the concentration of fluorescent ligand, and *K*_D_ is the calculated dissociation constant. Errors are the standard deviation of at least three experiments.

### Size exclusion chromatography

Mre11^HLH^-Rad50^NBD^ dimerization in the absence and presence of ATP (5 mM) was measured as previously described (23) via size exclusion chromatography (Superdex 200 Increase 10/300 GL column; GE Healthcare). Dimerization assays were performed at 4°C to limit ATP hydrolysis and maintain the dimer state. The area under the elution peaks were determined by box summation, and the fraction of dimerized Mre11^HLH^-Rad50^NBD^ was calculated by dividing the area for the dimer peak by the sum of the areas for the monomer and dimer peaks.

## RESULTS

### Rad50 D-loop cancer-associated mutants have increased ATP hydrolysis activity

**Figure 2.**
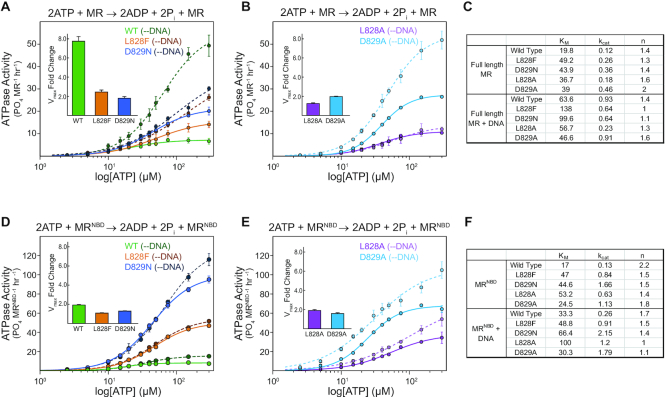
Rad50 D-loop mutations enhance ATP hydrolysis. (A and B) Steady-state ATP hydrolysis kinetics for wild type (green), L828F (orange), D829N (blue) (**A**), L828A (purple), and D829A (light blue) (**B**) full-length MR complexes in the absence (solid) and presence (dotted) of DNA. The data points and error bars are the average and standard deviation of three independent measurements, and the lines represent fits of the Hill equation to the data. *Inset* highlights the change in *V*_max_ upon addition of DNA. (**C**) Table of steady state kinetics constants (*K*_M_, μM; *k*_cat_, min^−1^; *n*, Hill coefficient) for MR mutants in the absence and presence of DNA. See Supplementary Table S1A and C for other kinetic constants and their associated errors. (D and E) Steady-state ATP hydrolysis kinetics for wild type (green), L828F (orange), D829N (blue) (**D**), L828A (purple), and D829A (light blue) (**E**) truncated MR^NBD^ complexes in the absence (solid) and presence (dotted) of DNA. The data points and error bars are the average and standard deviation of three independent measurements, and the lines represent fits of the Hill equation to the data. *Inset* highlights the change in *V*_max_ upon addition of DNA. (**F**) Table of steady state kinetics constants (*K*_M_, μM; *k*_cat_, min^−1^; *n*, Hill coefficient) for MR^NBD^ mutants in the absence and presence of DNA. See Supplementary Table S1B and D for other kinetic constants and their associated errors.

The ATP hydrolysis activity for wild type, L828F, and D829N D-loop mutant *Pf* Rad50 was measured in both full-length MR (Figure 2A, solid lines) and MR^NBD^ complexes (Figure 2D, solid lines) by the colorimetric detection of the inorganic phosphate product. Although the *K*_M_ for the mutants was ∼2.3-fold larger than wild type full-length *Pf* MR, the L828F and D829N mutations surprisingly showed faster ATP hydrolysis rates than wild type Rad50 (Figure 2C and Supplementary Table S1A). The catalytic efficiencies (i.e. *k*_cat_/*K*_M_) of L828F and D829N were similar- and ∼1.3-fold greater than wild type MR, respectively (Supplementary Table S1A). The effect of the disease-associated D-loop mutants was more pronounced for *Pf* MR^NBD^ complexes. Again, ∼2.5-fold increases were observed for the *K*_M_ values of both mutants, but significantly larger 6.5- and 12.8-fold increases were found for the ATP hydrolysis rates, which produced 2.2- and 4.7-fold greater catalytic efficiency for L282F and D829N, respectively (Figures 2D and F and Supplementary Table S1B). For both forms of the complex, the differences were not due to changes in the affinity for ATP as the *K*_D_ for substrate binding was similar for wild type and the mutants (Supplementary Figures S1A and B and Supplementary Table S1); instead, the differences in *k*_cat_/*K*_M_ were driven by the greater *k*_cat_.

We also examined the effect on ATP hydrolysis of alanine substitutions at each of the D-loop mutation sites (Figures 2B and E, solid lines). Generally, the L828A mutation yielded steady state ATP hydrolysis kinetics intermediate to wild type and L828F for both full-length MR and MR^NBD^ complexes (Figure 2 and Supplementary Tables S1A and S1B). In contrast, the D829A mutation caused significantly higher catalytic rates for both full-length (3.8-fold; Figure 2B and C and Supplementary Table S1A) and truncated Rad50s (8.7-fold; Figure 2E and F and Supplementary Table S1B), which resulted in the largest catalytic efficiencies of the D-loop mutants tested. Thus, these alanine mutations suggest that the universally conserved leucine and aspartate in the ABC ATP D-loop are critical for proper ATP hydrolysis activity in Rad50.

We then determined the steady-state hydrolysis activity in the presence of linearized DNA. As previously reported (21,22), we observed in the wild type enzyme an ∼8-fold increase in full-length MR and ∼2-fold increase in MR^NBD^ for the rate of ATP hydrolysis (Figures 2A and D, dashed lines and Supplementary Tables S1C and S1D). In contrast, ATP hydrolysis by the L828F and D829N mutants did not show the same stimulation in the presence of DNA: they had only 2.5- and 1.8-fold enhancement in rate within full-length MR or 1.1- and 1.3-fold enhancements in rate within MR^NBD^, respectively (Figures 2A and D, inset). The alanine mutants also showed less DNA stimulated ATP hydrolysis compared to wild type, especially in full-length MR (Figures 2B and E, inset).

In full-length MR complexes, wild type and the D-loop mutants displayed the characteristic cooperativity for ATP hydrolysis (Hill coefficient, *n* ∼ 1.4). Interestingly, the D829A mutation produced the largest Hill coefficient (n ∼ 2). Differences in cooperativity were more noticeable in MR^NBD^ complexes. Whereas wild type Rad50^NBD^ displayed cooperativity for ATP hydrolysis equal to the number of binding sites (*n* = 2.2), L828F, D829N, and L828A showed less cooperativity (*n* = 1.5). As was the case in full-length MR, MR^NBD^ D829A also had a higher level of cooperativity (*n* ∼ 1.8) than the other D-loop mutants. In all these cases however, the Hill coefficient >1 is consistent with the presence of a cooperative ‘inactive’ state in equilibrium with an ‘active’ state occurring after ATP-induced association of the NBDs but before ATP hydrolysis. The lower Hill coefficients for the mutants in MR^NBD^ suggest that they may disrupt this equilibrium within the closed conformation. In this framework of cooperativity, the DNA-stimulation of ATP hydrolysis activity could be the result of DNA binding acting as a positive allosteric regulator that shifts the equilibrium of the ‘inactive’ and ‘active’ states. In the presence of DNA, the Hill coefficient for full-length MR complexes remained ∼1.4, but a decrease in the Hill coefficient to ∼1.7 was seen for truncated MR^NBD^. Significantly smaller Hill coefficients were determined for the D-loop mutants in full-length MR in the presence of DNA with non-cooperative coefficients (*n* ∼ 1) observed for L828F and D829N MR complexes. Thus, for these two mutants adding DNA has completely uncoupled Rad50 NBD active sites; and they are no longer as sensitive to the ATP concentration as the wild type. Furthermore, the D-loop MR^NBD^ mutants showed no change or decreases in cooperativity in the presence of DNA, suggesting that the disease-associated mutations have already bypassed this step in allosteric regulation in the truncated construct. Together, these data provided strong primary evidence for the deregulation of Rad50 ATP hydrolysis in the D-loop mutants.

### Rad50 D-loop mutants form less ATP-induced closed state but equilibrate more rapidly

Given the unanticipated gain-of-function for ATP hydrolysis in the mutants, we next characterized ATP-induced Rad50 NBD association within the MR complex via Luminescence Resonance Energy Transfer (LRET) (38,39). Like fluorescence resonance energy transfer (FRET), LRET measures the distance dependent (r^−6^) transfer of energy from a donor to an acceptor. However, unlike FRET, LRET utilizes the long-lived luminescence of a chelated lanthanide (msec timescale) as the donor, which ensures that emission from direct acceptor excitation (nanoseconds timescale) does not contaminate the measurement. Among other advantages over FRET, LRET is sensitive to longer distances and is immune to problems associated with incomplete labeling (38,39). For these studies, chelated Tb^3+^ (donor) or Bodipy FL (acceptor) probes were attached to a cysteine in Rad50. Since the *Pf* Rad50 NBD does not contain a native cysteine residue, a single cysteine mutant (L51C) was introduced at a solvent exposed position. In the AMP–PNP-bound closed conformation of the MR complex, the L51Cs of the two Rad50 NBDs are ∼35–40 Å apart (30). This distance is near the Förster radius (*R*_0_ = 44.9 Å) of Tb^3+^ and Bodipy FL. The L51C mutation did not alter the structure, as judged by the high degree of overlap in NMR spectra, the ATP binding and hydrolysis activity of Rad50, or Mre11 nuclease activities (Supplementary Figure S2 and below). Note, full-length Rad50 has two cysteines at the apex of the Zn-Hook that coordinate a Zn^2+^ ion. Control labeling experiments on wild type full-length MR (i.e. L51) showed that these cysteines were labeled ∼10%; however, labeling at these positions had no effect on L51C LRET results, as they showed a very low level of donor-sensitized emission, and that fluorescence signal did not change upon ATP binding—likely because of the low labeling efficiency and the fact that the Zn-hook cysteines are too close together (∼4 Å) and therefore out of the sensitive range for an LRET pair with an R_0_ of 44.9 Å.

**Figure 3.**
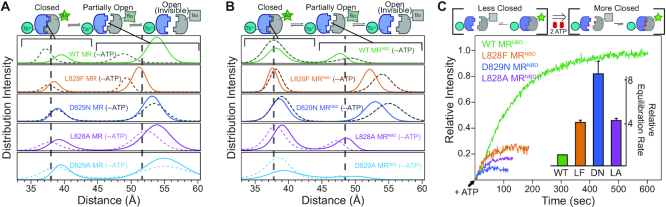
Rad50 D-loop mutations form less ‘closed’ state. (A and B) Distance distributions derived from exponential series method analysis of the LRET acceptor intensity decays. Distributions of the apo and ATP-bound states are shown for distances between Tb^3+^ (donor) and Bodipy FL (Bo; acceptor) labeled wild type (green), L828F (orange), D829N (blue), L828A (purple), and D829A (light blue) Rad50 NBDs in full-length MR (**A**) or truncated MR^NBD^ (**B**) complexes. Vertical dashed lines represent the average distance between donor and acceptor (from three measurements; see Table 1) of the closed and ‘partially open’ conformations for ATP-bound wild type MR. The schematic on top highlights that the closed (∼38 Å) and ‘partially open’ (∼48–55 Å) conformations are within the sensitive range of the LRET probes, while NBDs farther apart than ∼60 Å (i.e. open conformations) are invisible. (**C**) LRET time-based data where donor–sensitized acceptor intensity was measured after the addition of ATP to the indicated concentrations of MR^NBD^ complexes. The intensities for mutant curves are scaled by a factor of 1.5 for graphical purposes. *Inset* compares the equilibration rates relative to wild type MR.

In the absence of ATP, emission spectra for wild type full-length MR and MR^NBD^ complexes showed donor-sensitized acceptor fluorescence at ∼515 nm (Supplementary Figures S3A and S3C, respectively), indicating that for a population of MR complexes the two NBDs are in proximity for LRET. As expected, the presence of the coiled-coil domain in full-length MR resulted in more NBDs in proximity as signified by the greater fluorescence intensity at 515 nm as compared to MR^NBD^. Conversely, the D-loop mutants of each construct gave lower (full-length MR) or little-to-no emission (MR^NBD^) signal at 515 nm, denoting that either the overall donor–acceptor distance is longer (i.e. the NBDs are further apart) and/or the fraction of donor–acceptor pairs in proximity is smaller (i.e. less NBDs in the closed state; Supplementary Figures S3A and C). Next, donor–sensitized acceptor emission lifetime data was acquired to calculate the distance between the probes. Subsequent exponential series method analysis (29) and multiexponential fits of the LRET lifetime decays (Supplementary Figures S3B and D) provided distance distributions between the two Rad50 NBDs within MR complexes and ‘populations’ of molecules in each state (Figures 3A and B and Table 1). Note however, that reported populations from both fitting methods only reflect the number of molecules observed in the Bodipy FL lifetime measurements and will neglect states where NBDs are too far apart for donor-sensitized acceptor emission (including the possibility of molecules where Mre11s are dissociated). For wild type complexes in the absence of ATP, we observed a discrete closed (∼38–40 Å) conformation, consistent with the distances predicted from X-ray crystal structures (12–15), as well as a ‘partially open’ (∼48–54 Å) conformation. This data indicated that the MR complex samples the closed conformation in the absence of ATP. Moreover, as the fully open, ATP-free state traditionally observed in X-ray crystal structures (13) and the recently proposed dissociated form of the Mre11 dimer are invisible in the LRET experiment (i.e. the probes are too far apart for efficient energy transfer), this data revealed a dynamic equilibrium of at least three states (Figure 3A, top) as opposed to the two-state picture (i.e. open and closed, Fig 1A) provided by X-ray crystallography (12–15). Interestingly, the relative amount of full-length MR in the ‘partially open’ state is greater than what was observed for MR^NBD^. This difference likely results from the presence of the coiled-coil and Zn-hook domains shifting a population of molecules from the open to the ‘partially open’ state. Additionally, the distance between NBDs of the ‘partially open’ state is larger in full-length MR than MR^NBD^ in the absence of ATP, suggesting that the coiled-coil and Zn-hook domains may put a constraint on the orientation of the NBDs.

**Table 1.** Rad50 NBD distances determined by LRET

		‘Closed’			‘Partially open’	
		*R*_1_^a^	Molecules^b^		*R*_2_^a^	Molecules^b^
*Pf* Rad50		(Å)	(%)	Δ%	(Å)	(%)
A. Full-length MR LRET distances						
Wild Type	Apo	40.0 ± 0.5	15 ± 1	13	54.2 ± 0.5	85 ± 1
	ATP-Bound	38.0 ± 0.8	28 ± 3		51.8 ± 3.2	72 ± 3
L828F	Apo	39.1 ± 0.5	19 ± 2	6	51.6 ± 0.4	81 ± 2
	ATP-Bound	37.2 ± 2.1	25 ± 1		48.6 ± 2.8	75 ± 1
D829N	Apo	39.1 ± 0.3	19 ± 1	0	53.2 ± 0.6	81 ± 1
	ATP-Bound	38.3 ± 0.5	20 ± 1		51.9 ± 0.9	80 ± 1
L828A	Apo	39.8 ± 0.5	16 ± 1	0	53.7 ± 0.4	84 ± 1
	ATP-Bound	38.5 ± 0.8	17 ± 2		51.6 ± 1.3	83 ± 2
D829A	Apo	39.7 ± 1.3	17 ± 2	7	53.5 ± 1.1	83 ± 2
	ATP-Bound	38.0 ± 1.1	24 ± 1		51.7 ± 1.3	76 ± 1
B. MR^NBD^ LRET distances						
Wild Type	Apo	38.2 ± 0.5	52 ± 3	11	48.3 ± 1.7	48 ± 3
	ATP-Bound	37.8 ± 0.2	63 ± 2		48.5 ± 1.3	37 ± 2
L828F	Apo	38.6 ± 1.3	30 ± 2	0	54.3 ± 2.8	70 ± 2
	ATP-Bound	38.6 ± 0.4	30 ± 3		55.8 ± 2.6	70 ± 3
D829N	Apo	39.3 ± 0.6	33 ± 1	0	54.9 ± 1.2	67 ± 1
	ATP-Bound	38.8 ± 0.2	33 ± 1		55.4 ± 0.4	67 ± 1
L828A	Apo	38.0 ± 0.2	46 ± 6	8	46.0 ± 0.5	54 ± 6
	ATP-Bound	37.5 ± 1.2	54 ± 8		45.3 ± 1.8	46 ± 8
D829A	Apo	37.0 ± 0.9	42 ± 4	18	44.3 ± 1.1	58 ± 4
	ATP-Bound	37.2 ± 0.6	60 ± 9		43.3 ± 0.7	40 ± 9

^a^Distance (Å) between the Tb-chelate and Bodipy-FL probes attached to L51C on *Pf* Rad50 NBD. *R*_1_ is the second distance in a three-exponential fit of the Bodipy lifetimes, and *R*_2_ is the third distance of that fit. The first distance, which is result of the response from the instrument, is omitted from the analysis.

^b^Percentage of the signal that was in each distance, R, which were calculated from the amplitudes of the three-exponential fit and corrected for the fractional intensity contribution of each component $({}^{{{\rm Amplitude}_i}}/_{( {}^1/_{k_{{\rm Bodipy} - {\rm FL},\, {\rm i}}} - \, {}^1/_{k_{{\rm Tb}^{3 +}}})})$, where Amplitude_i_ and *k*_Bodipy-FL,i_ are the amplitude and rate of the second or third exponential in the fit, respectively, and k_Tb3+_ is the emission decay rate for the donor Tb-chelate). Note, this fraction only reports on LRET ‘visible’ molecules. Any molecules where the two probes are >∼60 Å apart are ‘invisible’ and thus do not contribute to the populations in this analysis.

Analysis of the donor–sensitized acceptor emission lifetime data revealed that the D-loop mutants also populate closed and ‘partially open’ conformations in the absence of ATP. In the full-length MR complex, the ‘partially open’ mutant complexes have about the same distance between the NBDs as wild type (Figure 3A, Supplementary Figure 4A, and Table 1). However, in MR^NBD^ the ‘partially open’ state of the L828F and D829N mutants is more open as compared to wild type (54 vs 48 Å) and are comparable to the ‘partially open’ states in full-length MR (Figure 3B, Supplementary Figure 4B, and Table 1). For many of the D-loop mutants, the exponential series method (Figure 3A and B) showed that broader distributions generally exist for the ‘partially open’ state when compared to wild type reflecting a larger range of conformations in this state. Consistent with the emission spectra (Supplementary Figure 3A), the LRET lifetime data revealed that in the absence of ATP, the full-length MR D-loop mutants had very similar populations in the closed state as compared to wild type MR. Conversely, in MR^NBD^, the populations of D-loop mutants in the closed state varied from 30% (L828F) to 46% (L828A) and were significantly smaller than the population of closed wild type MR^NBD^ (52%) (Figures 3A and B and Table 1).

As expected, the addition of saturating ATP to wild type MR shifted more molecules into the closed conformation as seen by the increase in the distribution intensity of the closed state (Figures 3A and B, dashed lines, and Table 1). In contrast, of the full-length D-loop mutants, only L828F and D829A showed moderate increases (∼50% of the change seen for wild type) in the population of the closed state upon ATP binding. In MR^NBD^, L828A showed a moderate increase, but adding ATP to D829A changed the population of the closed to more than wild type. We suggest that the changes in populations for MR^NBD^ are complicated by molecules coming from the ‘open’ state (as seen in SAXS) into the ‘partially open’ state. Not surprisingly, the distance between NBDs in the closed state remained constant in all of the ATP-bound complexes. Consistent with this data, size exclusion chromatography on isolated Rad50 samples revealed that <5% of D-loop mutant Rad50 NBDs converted to a stable dimer upon addition of ATP as compared to ∼30% for wild type (Supplementary Figure S1B). Thus, these results reveal a paradox for the D-loop mutations: increased ATP hydrolysis activity as compared to wild type, but a smaller relative population of the stable closed state from which ATP hydrolysis occurs.

To gain insight into the kinetics of ATP-induced Rad50 NBD association, we monitored the increase in LRET signal intensity over time after the addition of saturating ATP. These experiments provide kinetic information on Rad50 NBD association and dissociation by measuring the time required to reach a new open-to-closed equilibrium in response to Mg⋅ATP binding (Figure 3C, top). As expected from the population and lifetime measurements, the LRET intensity for the wild type MR^NBD^ complex plateaued at a higher value than that of the L828F, L828A and D829N mutants, reflecting the greater population of ATP-induced closed state. The equilibration rate constants for ATP-induced open-to-closed equilibrium of full-length (80.3 s^−1^) and truncated MR (74.1 s^−1^) were similar. Unfortunately, we were only able to measure equilibrium kinetics in full-length MR for the wild type complex, as the mutants did not have sufficient change into the closed conformation or reached the new ATP-bound equilibrium too quickly to measure. Remarkably, in MR^NBD^ we observed an increased equilibration rate in the mutants, where L828F, D829N, and L828A had ∼4-, ∼8- and ∼4-times faster approach to equilibrium than wild type, respectively (Figure 3C, inset). This rate was even faster for D829A MR^NBD^, and it reached the new equilibrium too quickly to accurately determine a rate. Under the conditions used here, the equilibration rate is the sum of the Mg⋅ATP-bound Rad50 association and dissociation rates; therefore, the faster approach to equilibrium that we observe could be due to an increase in either rate or in both rates. If only the association rate increased for the D-loop mutants, we would have observed a larger population of the closed state. Therefore, given the lower populations of closed MR in the mutants (Figure 3), these increased equilibration rates are likely the result of faster Rad50 NBD dissociation. A strong correlation between Rad50 equilibration rates in MR^NBD^ and ATPase maximum velocities (*R*^2^ = 0.975; Supplementary Figure S4C) suggests that Rad50 NBD dissociation is directly linked to the observed rate of ATP hydrolysis.

### Mutations to the D-loop have disparate effects on Rad50 structure and dynamics

**Figure 4.**
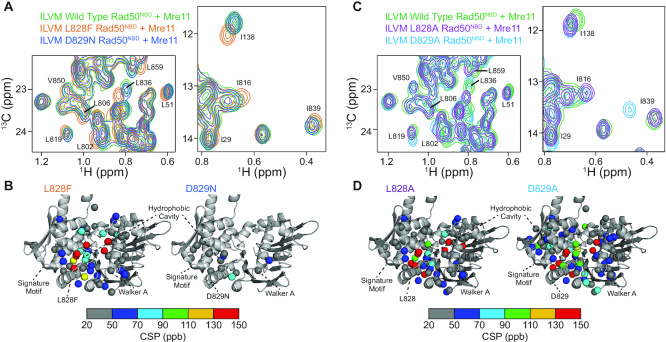
D-loop mutations disparately affect Rad50 structure. (**A**) Regions of the overlaid 2D ^13^C,^1^H HMQC spectra for U-^2^H^12^C, Ileδ1–^13^CH_3_, Leuδ/Valγ-[^12^CD_3_/^13^CH_3_], Metϵ-^13^CH_3_ (ILVM)-labeled wild type (green), L828F (orange) and D829N (blue) Rad50^NBD^ bound to unlabeled Mre11 (see Supplementary Figure S5 for the full spectra). The labeled peaks experience a chemical shift perturbation (CSP) indicating a change in structure upon D-loop mutation. (**B**) Methyl group resonances that have a significant CSP are mapped on the structure of Rad50 (pdb: 3QKS (15)) as spheres. CSPs in the L828F and D829N mutants are shown on the left and right structures, respectively, and site of each mutation is depicted with sticks. Methyl groups with small (<20 ppb) or no CSP are hidden. The color of the sphere indicates the relative structural change, as defined by the gradient below the structures. (**C**) Regions of the overlaid 2D ^13^C,^1^H HMQC spectra for ILVM-labeled wild type (green), L828A (purple), and D829A (light blue) Rad50^NBD^ bound to unlabeled Mre11 (see Supplementary Figure S5 for the full spectra). The labeled peaks experience a chemical shift perturbation (CSP) indicating a change in structure upon D-loop mutation. (**D**) Methyl group resonances that have a significant CSP are mapped on the structure of Rad50 (pdb: 3QKS (15)) as spheres. CSPs in the L828A and D829A mutants are shown on the left and right structures, respectively, and site of each mutation is depicted with sticks. Methyl groups with small (<20 ppb) or no CSP are hidden. The color of the sphere indicates the relative structural change, as defined by the gradient below the structures.

To obtain higher resolution structural information, methyl-based NMR spectroscopy (34,40) was employed on the ∼180 kDa MR^NBD^ complexes. We used uniformly deuterated, side chain methyl group Ileδ1-[^13^CH_3_], Leuδ/Valγ-[^13^CH_3_,^12^CD_3_], Metϵ-[^13^CH_3_] (ILVM)-labeled wild type, L828F, D829N, L828A and D829A *Pf* Rad50^NBD^ in complex with unlabeled Mre11. The ILVM methyl group assignments we previously determined on isolated Rad50 (23) readily transferred to the MR^NBD^ complex. Spectral overlays of the wild type, L828F, and D829N two-dimensional ^13^C,^1^H heteronuclear multiple quantum coherence (HMQC) correlation spectra (Figure 4A and Supplementary Figure S5) revealed that L828F had large chemical shift perturbations (CSPs) around the site of the mutation, in the adjacent hydrophobic cavity, and in other regions that spanned up to ∼50 Å away from the site of mutation. These long range CSPs to the Q-loop, DNA binding, and Walker A motifs indicated that the previously described Rad50 allosteric network (15,23,24) is affected upon L828F mutation (Figure 4B, left). Conversely, D829N only resulted in minor CSPs local to the D-loop and to residues in the Rad50-Rad50 binding interface (Figure 4B, right). Spectral overlays of the wild type and L828A HMQC correlation spectra (Figure 4C and Supplementary Figure S5) again showed CSPs around the site of the mutation and to regions of Rad50^NBD^ extending from the hydrophobic cavity (Figure 4D, left), like L828F. Unlike D829N, the D829A mutation produced the largest, most widespread CSPs in Rad50^NBD^ (e.g. L836, L819 and I839 in Figure 4C), and as is the case for the L828F and L828A mutants, these CSPs extend to other regions in the NBD that span ∼50 Å away from the site of the mutation and affect regions important for ATP binding and hydrolysis. However, in contrast to the L828 mutants, the D829A mutation also caused CSPs to M808, a hinge residue that is a reporter on allostery in the hinge region (23). Note, the peak for M808 does not does not shift for any of the other D-loop mutants; therefore, it appears that the D829A mutation also affects the allosteric network around the hinge, and this difference may contribute to its departure from the other D-loop mutants in biochemical assays (e.g. Hill coefficients and exonuclease activity, see below).

**Figure 5.**
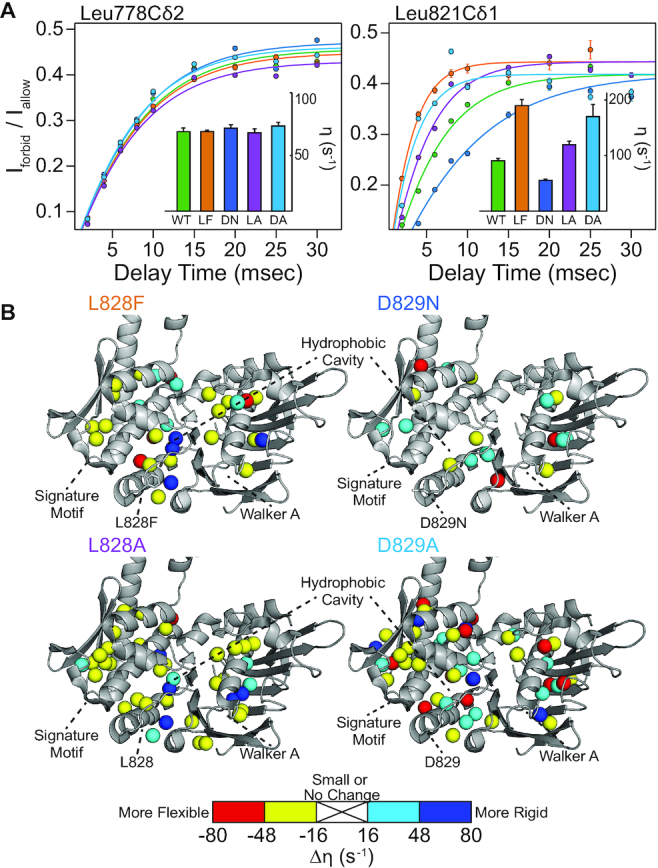
Mutations to the D-loop alter the dynamic landscape of Rad50 NBD. (**A**) Representative build up curves for the ratio of intensities (*I*_forbid_/*I*_allow_) from methyl group ^1^H triple quantum ‘forbidden’ experiments. Data are presented for Leu778Cδ2 (left) and Leu821Cδ1 (right) in wild type (green), L828F (orange), D829N (blue), L828A (purple), and D829A (light blue) (see Supplementary Figure S6 for other curves). Error bars were derived from the noise of the spectra, and solid lines are fits to the experimental data. *Insets* show bar charts of the calculated intra-methyl ^1^H–^1^H dipolar cross-correlated relaxation rate (η). Leu778Cδ2 (left) is a methyl group that does not have a significant change in the amplitude of methyl group dynamics upon mutation. In contrast, Leu821Cδ1 (right) has major changes in flexibility for L828F (decrease) and D829N (increase). (**B**) Methyl groups experiencing a change of dynamics upon L828F (top left), D829N (top right), L828A (bottom left) and D829A (bottom right) mutation (Δη = η_mut_ – η_wt_) are indicated as spheres on the crystal structure of Rad50 (pdb: 3QKS (15)). Methyl groups with small or no change of dynamics (|Δη| < 16 s^−1^) are hidden. The color of the sphere indicates the relative dynamics change (see scale under structures).

Altered molecular motions have been linked to changes in enzymatic activity in many systems including Rad50 (23,41,42). Thus, we also investigated the amplitude of picosecond-to-nanosecond timescale dynamics via the ‘forbidden’ methyl triple quantum ^1^H–^1^H dipolar cross-correlated relaxation rate (η; Figure 5A) (37). *η* rates are directly proportional to the product of global macromolecular tumbling and the local amplitude of picosecond-to-nanosecond timescale side chain methyl group dynamics (37). In our analysis, we assumed that global tumbling is the same for wild type and D-loop mutant MR^NBD^ complexes. Thus, changes in *η* rates upon mutation (Δη = η_mut_ – η_wt_) reflected changes in the amplitude of local methyl group dynamics. L828F had many large changes in side chain methyl group dynamics throughout the protein, again revealing that the dynamic allosteric network is affected (Figure 5B, top left, and Supplementary Figure S6). As with the CSPs, the hydrophobic cavity of L828F had the largest changes to η and was generally more flexible than wild type; furthermore, there are residues neighboring the cavity that rigidify. As side chain methyl group dynamics are a reporter of conformational entropy (43), this juxtaposition suggested some entropy compensation from the next layer of amino acids. D829N had fewer and more dispersed changes in dynamics (Figure 5B, top right, and Supplementary Figure S6), which mimicked the limited changes in chemical environment shown by CSPs. As was the case for the CSPs, L828A had very similar changes to L828F in η, which led to generally more flexibility (i.e. increases in η) in the hydrophobic cavity and the signature motif (Figure 5B, bottom left, and Supplementary Figure S6). Yet, the changes in η are slightly less than those seen for L828F, mirroring the same intermediate effect of the L828A mutation observed for ATP hydrolysis and LRET data. Lastly, the D829A mutant showed the largest changes to η, which affected much more of the NBD than D829N did (Figure 5B, bottom right, and Supplementary Figure S6). Like the mutations to L828, the changes in dynamics were also to the hydrophobic cavity and other regions within the NBD; however, the D829A mutation resulted in more wide spread changes and both increases and decreases in η. Thus, our NMR data revealed that the L828 and D829 mutations had different effects on the local structure and dynamics of Rad50 NBD. However, all of the mutations lead to a smaller population of the MR closed state and enhanced ATP hydrolysis activity.

### Rad50 D-loop mutations affect the nuclease activity of the MR complex

**Figure 6.**
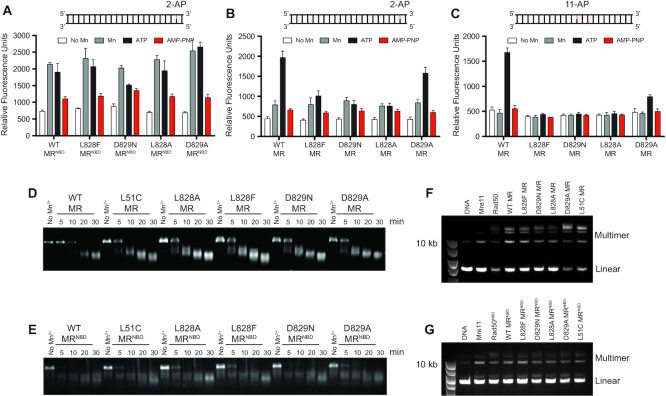
D-loop mutations affects the Mre11 nuclease activity. (A and B) Mre11 exonuclease activity for truncated MR^NBD^ (**A**) and full-length MR (**B**) complexes with various Rad50 mutations. Exonuclease activity was determined with a 29-nt dsDNA containing a 2-AP probe at the second nucleotide from the 3′-end (Exo2) in the presence of 5 mM Mg^2+^ (white bars); 1 mM Mn^2+^ (gray bars); 1 mM Mn^2+^, 5 mM Mg^2+^ and 1 mM ATP (black bars); or 1 mM Mn^2+^, 5 mM Mg^2+^ and 1 mM AMP-PNP (red bars). Bars and error bars represent the average and standard deviation of three replicates. (**C**) Mre11 exonuclease activity for full-length MR complexes with various Rad50 mutations. Exonuclease activity was determined with a 29-nt dsDNA containing a 2-AP probe at the eleventh nucleotide from the 3′-end (Exo11) in the presence of 5 mM Mg^2+^ (white bars); 1 mM Mn^2+^ (gray bars); 1 mM Mn^2+^, 5 mM Mg^2+^, and 1 mM ATP (black bars); or 1 mM Mn^2+^, 5 mM Mg^2+^ and 1 mM AMP-PNP (red bars). Bars and error bars represent the average and standard deviation of three replicates. (D and E) Mre11 endonuclease activity for full-length MR (**D**) and MR^NBD^ (**E**) complexes with various Rad50 mutations. Endonuclease activity was measured using the ΦX174 single-stranded plasmid DNA in the absence (No Mn^2+^) or presence of 1 mM Mn^2+^, 5 mM Mg^2+^ and 1 mM ATP. (F and G) DNA end tethering assay for the various mutants in MR (**F**) and MR^NBD^ (**G**) complexes. End tethering was assessed with linearized plasmid DNA in the presence of 2 mM ATP.

We have previously shown in MR^NBD^ that mutations to the Rad50 hinge region, which couples the coiled-coil domain to the ABC ATPase signature motif, also had significantly increased ATP hydrolysis activity (23). Interestingly, these hinge mutations increased the Mre11 exonuclease activity of the MR^NBD^ complex, but only in the presence of ATP: without ATP, they essentially cleaved like wild type. The changes in Rad50 ATP hydrolysis activity were strongly correlated with this increase in Mre11 exonuclease activity in the presence of ATP. Thus, we next determined the Mre11 exonuclease activity on the same dsDNA substrate (Exo2 DNA, where a 2-aminopurine (2-AP) reporter is two nucleotides in from the 3′-end of the duplex) in full-length MR and MR^NBD^ complexes made with the Rad50 D-loop mutants. As shown in Figure 6A, we did not observe any significant differences in the Mn^2+^-dependent exonuclease activity of the MR^NBD^ D-loop mutants when compared to the wild type complex (gray bars). Moreover, in the presence of ATP, the activity did not change significantly except for the D829N complex, which had reduced activity (black bars). This is in contrast to the Exo2 nuclease data for the MR^NBD^ hinge mutants, where higher ATP hydrolysis activity was correlated with high exonuclease activity. As expected, the addition of the non-hydrolyzable nucleotide analog AMP-PNP, which stabilizes the complex into the closed state and occludes the Mre11 active site, led to decreased activity for all the MR^NBD^ complexes (red bars). An important difference between the mutants in the Rad50 hinge region and the D-loop mutants is the effect of mutation on Rad50 ATP-induced dimerization: the hinge mutants led to an increase in the population of the stable Rad50 dimer (1.5–2.0-fold increase as detected by size exclusion chromatography), while the D-loop mutants resulted in a dramatic decrease in Rad50 dimer (∼6-fold decrease). These data suggest that the ability to form an associated Rad50 is related to Mre11 exonuclease activity in the MR complex.

For full-length MR complexes, we observed only a low level of Mn^2+^-dependent exonuclease activity on the Exo2 substrate for wild type and the D-loop mutants in the absence of ATP (Figure 6B, gray bars). This activity is comparable to what was observed in the presence of AMP-PNP (red bars). Upon addition of ATP, a clear increase in exonuclease activity is seen for wild type MR, but not for L828F, D829N and L828A (black bars). Unlike the other D-loop mutations, D829A showed ATP-dependent exonuclease activity. The lack of exonuclease activity in L828F, D829N and L828A was even more obvious when we used a substrate where the 2-AP reporter is eleven nucleotides from the 3′-end of the double-stranded DNA (Exo11). Full-length MR exonuclease activity was also ATP-dependent for this substrate, and none of the D-loop mutants displayed activity greater than the no Mn^2+^ or AMP–PNP controls (Figure 6C), again, with the exception of the D829A mutant. When compared to full-length MR, which requires ATP, the MR^NBD^ complexes exhibited exonuclease activity to at least the second nucleotide, where the Exo2 probe is located, in the absence of ATP. Notably, the MR^NBD^ complexes are not active on the Exo11 substrate (data not shown). Thus, the coiled-coil and Zn-hook domains alter the architecture of the Mre11 active site, through different orientations of the Rad50 NBDs, which leads to ATP-sensitive nuclease activity.

We next examined the effect of Rad50 D-loop mutations on the single-stranded DNA endonuclease activity of Mre11. Both full-length MR (Figure 6D) and MR^NBD^ (Figure 6E) complexes cleave a circular single-stranded virion DNA plasmid in the presence of Mn^2+^ and ATP, which was visualized as smaller fragments of the DNA migrating further in an agarose gel. Surprisingly, all of the D-loop mutations appeared to have similar or increased endonuclease activity in both forms of the complex. Thus, the increased ATP hydrolysis rate and decreased stability of the MR closed state, stemming from the increased rate of NBD dissociation, appears to affect the nuclease activities of MR differently: decreasing the exonuclease while perhaps even increasing the endonuclease.

Lastly, we determined the capability of the Rad50 D-loop mutations to tether the ends of broken double-stranded DNA. Full-length MR and truncated MR^NBD^ complexes were added to linearized plasmid DNA in the presence of ATP. Tethering activity was then assessed by the ability of ATP-dependent T4 DNA ligase to join the adjacent broken ends (18). Full-length Rad50, and to a much lesser extent Mre11, is capable of facilitating the ligation linear DNA into higher order DNA products, as previously reported (18) (Figure 6F). Consistent with our LRET data, which showed a constant population of closed-state MR complexes (Figure 3), the D-loop mutants also produced ligated products in the presence of ATP. In fact, the intensity of the higher order bands mirrored the populations we observed in LRET, with L828F and D829A yielding similar results as wild type and D829N and L828A less. Ligated DNA products were also visible in truncated MR^NBD^ complexes (Figure 6G); however, the ligation products were less. Therefore, the coiled-coil and Zn-hook domains promote end-tethering activities, which occurs through their DNA binding ability (18).

## DISCUSSION

**Figure 7.**
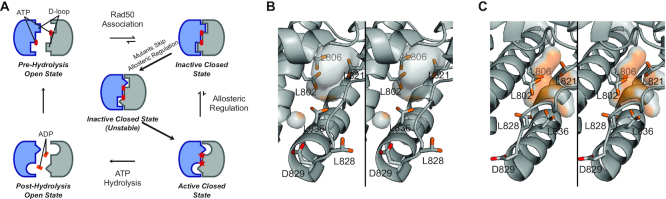
Perturbation of the D-loop circumvents Rad50 allosteric regulation. (**A**) Cartoon representation of the proposed steps required for Rad50 ATP hydrolysis. Rad50 NBDs within an MR complex are shown in blue and gray. Structural changes upon D-loop mutation are indicated as different shapes (i.e. square, triangle, and half-circle). ATP: red ovals; ATP in the hydrolysis competent state: red stars; ADP: orange ovals. (**B**) Stereo view of the structure of the D-loop for *Pf* Rad50 NBD in the ATP-free open state (pdb: 3QKS (15)). D828, L828 and methyl-containing residues that line the hydrophobic cavity are shown as sticks. The hydrophobic cavity is outlined in the semi-transparent surface. (**C**) Stereo view of the structure of the D-loop for *Pf* Rad50 NBD in the ATP-bound closed state (pdb: 3QKT (15)). Highlighted residues and the hydrophobic cavity are depicted as in (B).

Figure 7A describes our model for the structural and dynamic mechanism of ATP hydrolysis in wild type Rad50: (i) ATP binding, (ii) Rad50 NBD association, (iii) cooperative structure change subject to allosteric regulation and (iv) ATP hydrolysis and Rad50 NBD and product (ADP + Pi) dissociation. With a *K*_D_ for ATP of ∼3–5 μM, cellular concentrations of ATP (∼1–10 mM) produce fully bound Rad50⋅Mg⋅ATP, therein promoting Rad50 NBD association into a population of closed and ‘partially open’ states. Based on crystal structures of AMP-PNP-bound closed *Pf* Rad50 NBD (15), D829 (human D1237) in the D-loop forms an interaction with the Walker A motif of the *trans*-Rad50 NBD (Figures 1 and 7A). We hypothesize this linchpin interaction is required to form the stable closed state and transmit the cooperative structural changes required to allosterically regulate MR ATP hydrolysis. Consistent with this idea, recent molecular dynamics simulations revealed a stable ATP-bound, closed state of MR from which allosterically modulated cooperative hydrolysis could occur (42). Furthermore, it has been found that the D-loop – Walker A interaction of the ABC transporter associated with antigen processing, TAP, was necessary to couple unidirectional peptide substrate transport with ATP hydrolysis (44). Thus, we propose that the Rad50 D-loop–Walker A interaction in MR increases the population of an ‘inactive’ closed state, resulting in slow ATP hydrolysis. Rad50 is a notoriously slow ATPase (18,45), and wild type *Pf* MR only performs ATP hydrolysis once every ∼8.3 min *in vitro*; however, we and others show that the rate of hydrolysis increases in the presence of DNA (21, 22). Thus, DNA binding likely acts as an allosteric activator increasing the population of an ‘active’ closed state. ATP hydrolysis and Rad50 NBD dissociation then occur from this ‘active’ closed state.

The enhanced ATP hydrolysis rates, decreases in cooperativity, lower populations of the ATP-bound MR stable closed state, and the likely faster Rad50 NBD dissociation all suggest that the rate limiting step of Rad50 cooperative structural changes is being circumvented in these two D-loop mutants. We show that L828F reorients the structural and dynamic landscape within a Rad50 protomer and therefore hypothesize that L828F shifts the ‘inactive’ to ‘active’ closed state equilibrium toward the ATP hydrolysis competent ‘active’ form. Ile138, Ile143, Leu802 and Leu836 methyl groups, which line the hydrophobic cavity (Figure 7B), experience the largest CSPs; therefore, we think that the mutated F828 side chain alters the orientation of the D-loop resulting in a conformation of the hydrophobic cavity and D-loop that mimics the ATP-bound ‘active’ form, pre-emptively orienting the Rad50 active sites for ATP hydrolysis, and prohibiting the formation of the stable ‘closed’ Rad50 NBDs (Figure 7A). The importance of the hydrophobic cavity in Rad50 was previously shown with structural and functional studies of a L802W (*Pf* Rad50) mutation. This mutation, which puts a bulky indole side chain into the top of the hydrophobic cavity, also displayed an increase in *k*_cat_ for ATP hydrolysis and decrease in ATP-induced Rad50 association (18). Moreover, the L802W mutation resulted in increased mobility of the D-loop, as judged by crystallographic temperature-factors, in line with our NMR data connecting the D-loop and hydrophobic cavity in L828F and our model for the D-loop forming a linchpin interaction. Finally, cooperativity in L828F is reduced between active sites because each are already primed for catalysis. As such, the mutant is not as sensitive to allosteric modulation by DNA. In contrast to L828F, D829N does not show significant structural or dynamics changes, suggesting a different mechanism for circumventing allosteric regulation. As the D-loop holds Rad50 in the ‘inactive’ state, we propose that the D829N mutation only weakly forms the Walker A–D-loop interaction resulting in unstable ‘inactive’ closed Rad50. This idea is consistent with an aspartate to alanine mutation in the TAP ABC ATPase: there, the D-loop mutation uncoupled unidirectional transport of substrate peptide from ATP hydrolysis, and the ATP hydrolysis activity of the mutation was not stimulated by peptide (44), similar to what was observed here. Thus, the weak D-loop interaction in Rad50 D829N allows the enzyme to readily proceed to ATP hydrolysis bypassing the cooperative structural change (Figure 7A). Since the ‘inactive’ closed Rad50 is destabilized, cooperativity is also reduced between active sites of D892N, and the mutant is also not sensitive to stimulation by DNA.

Generally, compared to L828F, the L828A mutation was not as active in ATP hydrolysis, not as stimulated by DNA, and had similar response to ATP-induced dimerization (i.e. did not appreciably shift the population to the closed state). L828A produced changes in structure and dynamics of the NBD to the same regions as the L828F mutation. Thus, this mutation also alters the conformation of the D-loop and nearby hydrophobic cavity. We propose therefore that the alanine mutation acts in the same way as the L828F mutation: changing the conformation of the D-loop such that the stable ‘closed’ of the enzyme cannot form. Finally, the D829A mutation had the highest level of ATP hydrolysis in the absence of DNA and forms the broadest distribution of ‘partially open’ state. Moreover, the D829A mutation had the largest and most widespread changes to the Rad50 NBD structure and dynamics. In the wild type structure, D829 might acts as a helical N-capping residue (Figures 1 and 7B). Mutation to asparagine does not significantly alter free energy for N-capping, but mutation to alanine does (46). Altered stabilization of this alpha helix could lead to the wide spread changes in structure and dynamics that we see for this mutant. Further, the alanine mutation completely removes all hydrogen bond accepting groups from the conserved aspartate, in contrast to the asparagine mutant that can still accept a hydrogen bond on the carbonyl group, which would disrupt the interaction with the *trans*-Walker A motif. Thus, we propose that the changes to alpha helix capping leads to an even weaker D-loop interaction in D829A bypassing the rate-limiting cooperative structural change in Rad50.

We have previously characterized the structure, dynamics, Rad50 ATP hydrolysis, and Mre11 exonuclease activity for a number of mutations to the hinge region of *Pf* Rad50 (R805E, V156M and V160M) – a motif that in the ATP-free state is stabilized by an ionic interaction between the side chain of basic switch residue R805 and the backbone carbonyl of N134 (15,18,23). The hinge region packs against the extended signature helix, which leads to the signature motif, one of the two contact points in the Rad50 closed state. We found that hinge mutations that disrupted the basic switch led to increases in side chain methyl group dynamics (primarily along the extended signature helix), ATP-induced Rad50 association, Rad50 ATP hydrolysis, and Mre11 exonuclease activity (23). Based on our data, we proposed that these mutations revealed a dynamic state that is on pathway to the ATP-bound Rad50 closed state (i.e. NBD Association in Figure 7A). In the context of our model here, the weakening of the basic switch interaction by the hinge mutations would result in greater flux through the inactive closed state leading to an increased population of the active closed state and more ATP hydrolysis. We propose that the cancer-associated mutations within the D-loop, the second contact point in the Rad50 closed state, modulate the linchpin Walker A–D-loop interaction that is critical for cooperativity and allosteric regulation associated with ATP hydrolysis (i.e. Allosteric Regulation in Figure 7A). In contrast to the hinge mutants, disrupting the D-loop resulted in changes in side chain methyl group dynamics in the hydrophobic cavity (for L828F and L828A), less ATP-induced Rad50 association, and a loss of Mre11 exonuclease activity. Interestingly, we still observed some exonuclease activity for D829A. Unlike the other D-loop mutants, D829A perturbed the side chain methyl group resonances of hinge residue M808, suggesting that this mutation not only perturbs the D-loop interactions but also alters the allosteric network through the hinge region. We think the remaining exonuclease activity is a result of the combinatorial effect of changing both points (i.e. the signature motif and D-loop–Walker A interaction, Figure 1B) of NBD association. Overall, our data implies that the signature motif and D-loop interactions separately control Rad50 activity, since disrupting either contact point in the closed state leads to greater ATP hydrolysis.

Initial characterization of these two Rad50 D-loop mutants in *S. cerevisiae* and mouse embryonic fibroblasts revealed that mutant MRN complexes were deficient for downstream Tel1/ATM signaling (26). The ATP-bound, closed conformation of MRN is partially responsible for activating ATM (42,47); thus, our results, which show only a low level of stable NBD association in L828F and D829N Rad50, explain this *in vivo* observation. Moreover, it was found that yeast carrying the Rad50 D-loop mutations had shorter than wild type telomeres (26). Other mutations in the ATP-bound, closed Rad50 DNA binding region also produced the same phenotype (16), highlighting the importance of this function in telomere length maintenance. The low population of the closed state we observed here for the D-loop mutants can also explain the shorter telomeres. The clinical trial that identified the human Rad50 L1237F mutant and the subsequent characterization of the D-loop mutants utilized a topoisomerase I inhibitor, which generates ssDNA breaks with attached topoisomerase adducts on the 3′-phosphate. However, we noted that MR endonuclease activity, which is thought to be important in the removal such adducts (48), is unaffected by these mutations. Our observation is nevertheless consistent with recent results in *Xenopus* extracts demonstrating that although MRN is required for removal of bulky 3′ end adducts, Mre11 nuclease activity is not (49). Lastly, we tested the ability of D-loop mutant MR complexes to facilitate end tethering, as determined by the ability of T4 DNA ligase to join the ends of linear DNA. For both the full-length MR and truncated MR^NBD^ wild type and mutated D-loop complexes, we observed ligation products indicative of end tethering activity. Previous results on the *Pf* Rad50 L802W mutant, which does not form ATP-bound stable dimer, showed that that mutant is capable of DNA end tethering (18). Moreover, it has also been reported that the signature motif mutant yeast S1205R (*Pf* S793R), which does not dimerize or hydrolyze ATP, can also perform DNA end tethering; whereas, a Walker A mutant, which can dimerize but not hydrolyze ATP cannot (50). Thus, Rad50 ATP-induced dimerization (or ATP hydrolysis for that matter) is not required for end tethering activity.

It was also shown in yeast that D-loop leucine to alanine or arginine mutations did not produce the same magnitude of response as the phenylalanine substitution *in vivo* (26), consistent with our results: in that study, the L828A homolog also had an intermediate effect in biochemical and biophysical assays when compared to the leucine mutation. Lastly, it was demonstrated in yeast that the aspartate to alanine mutation produced an attenuated response *in vivo*: for example, the aspartate to alanine mutation was much less sensitive to genotoxic agents than the asparagine mutant (26). Based on our side chain methyl group CSPs, the *Pf* D829A mutation affects the hinge region allosteric network possibly leading to a mixed phenotype between the Mre11 exonuclease dead and ATP-dependent exonuclease active which could give rise to the less severe defects of this mutation in yeast. As a side note, we believe this agreement in the nuanced results between our model system *Pf* mutants and *in vivo* yeast mutants gives further credence to using archaebacteria to study MR.

MRN expression has been linked to reduced progression-free survival in patients with colorectal cancers (51), and the knockdown of Rad50 sensitizes colorectal cancer cell lines to ionizing irradiation (52); additionally, a Rad50 mutant lacking the NBDs sensitizes nasopharyngeal carcinoma to irradiation (53) highlighting the potential for allosteric inhibitors of Rad50. Interestingly, previous modeling of small molecule activators that bind to the NBDs of the related Cystic Fibrosis Transmembrane Conductance Regulator (CFTR) revealed a series of molecules that bind in the region of the hydrophobic cavity (54), which suggests that a similar molecules could be found for Rad50 (18). Based on the results reported here, we anticipate that pharmacologically mimicking the L828F structural/dynamic state with a small molecule could result in synthetic lethality in cancer cells when combined with DNA damage inducing agents, such as topoisomerase I inhibitors, and a checkpoint kinase (CHK1/2) inhibitor, as MRN would be improperly regulated and downstream ATM signaling would be disrupted.

## DATA AVAILABILITY

Side chain methyl chemical shifts for wild type (BMRB ID: 27955), L828F (27956), D892N (27957), L828A (28049) and D829A (28051) are available from the Biological Magnetic Resonance Bank. All other data used in this study are available within the article, the Supplementary files, or from the authors upon request.

## Supplementary Material

gkz1228_Supplemental_File
