# Supplementary material for: Adjacent mutations in the archaeal Rad50 ABC ATPase D-loop disrupt allosteric regulation of ATP hydrolysis through different mechanisms
Source: Nucleic Acids Res. 2019 Dec 31;48(5):2457–72. doi: 10.1093/nar/gkz1228 (PMC7049730; doi:10.1093/nar/gkz1228)
Supplement: gkz1228_Supplemental_File [file gkz1228_supplemental_file.pdf]

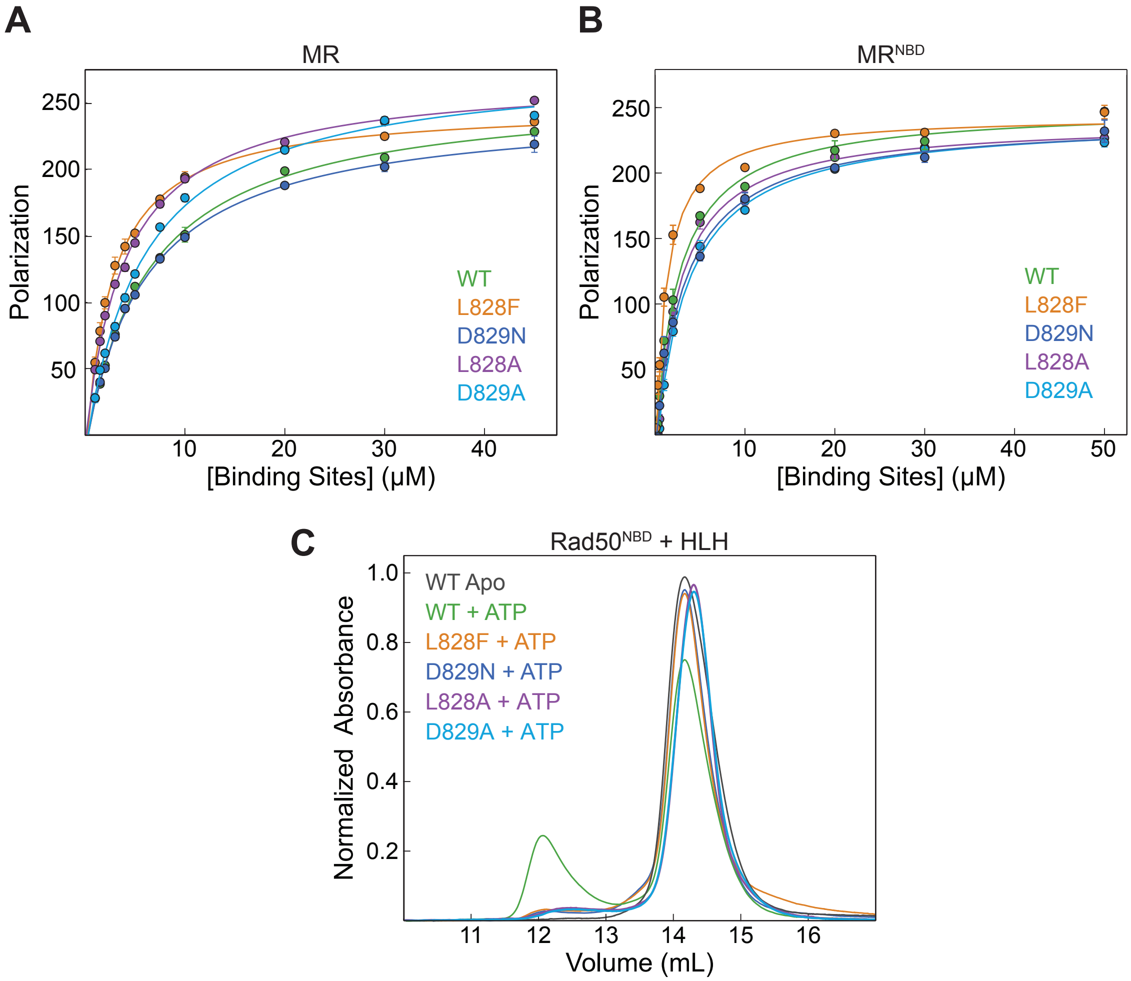

Supplementary Figure 1. Rad50 ATP binding and protomer association. (**A** and **B**) Bodipy FL-labeled ATP (5 nM) was titrated with wild type and D-loop mutant full-length MR (**A**) and MR^{NBD} (**B**) complexes. Fluorescence polarization values (Ex. 485 nm/Em. 530 nm) for wild type (green), L828F (orange), D829N (blue), L828A (purple), and D829A (light blue) were fit to the quadratic form of the simple two-state binding equation to obtain binding affinities (solid line). Data points and error bars are the average and standard deviation of three replicates. $K_{D,ATP}$ values from the fits are reported in Supplementary Table 1. (**C**) The ATP-dependent dimerization of Rad50 nucleotide binding domain (NBD), in complex with the Mre11 C-terminal helix-loop-helix motif, was observed by size exclusion chromatography. Wild type, L828F, D829N, L828A, and D829A chromatograms in the presence of ATP are reported as green, orange, blue, purple, and light blue lines, respectively. Wild type in the absence of ATP is shown as a grey line.

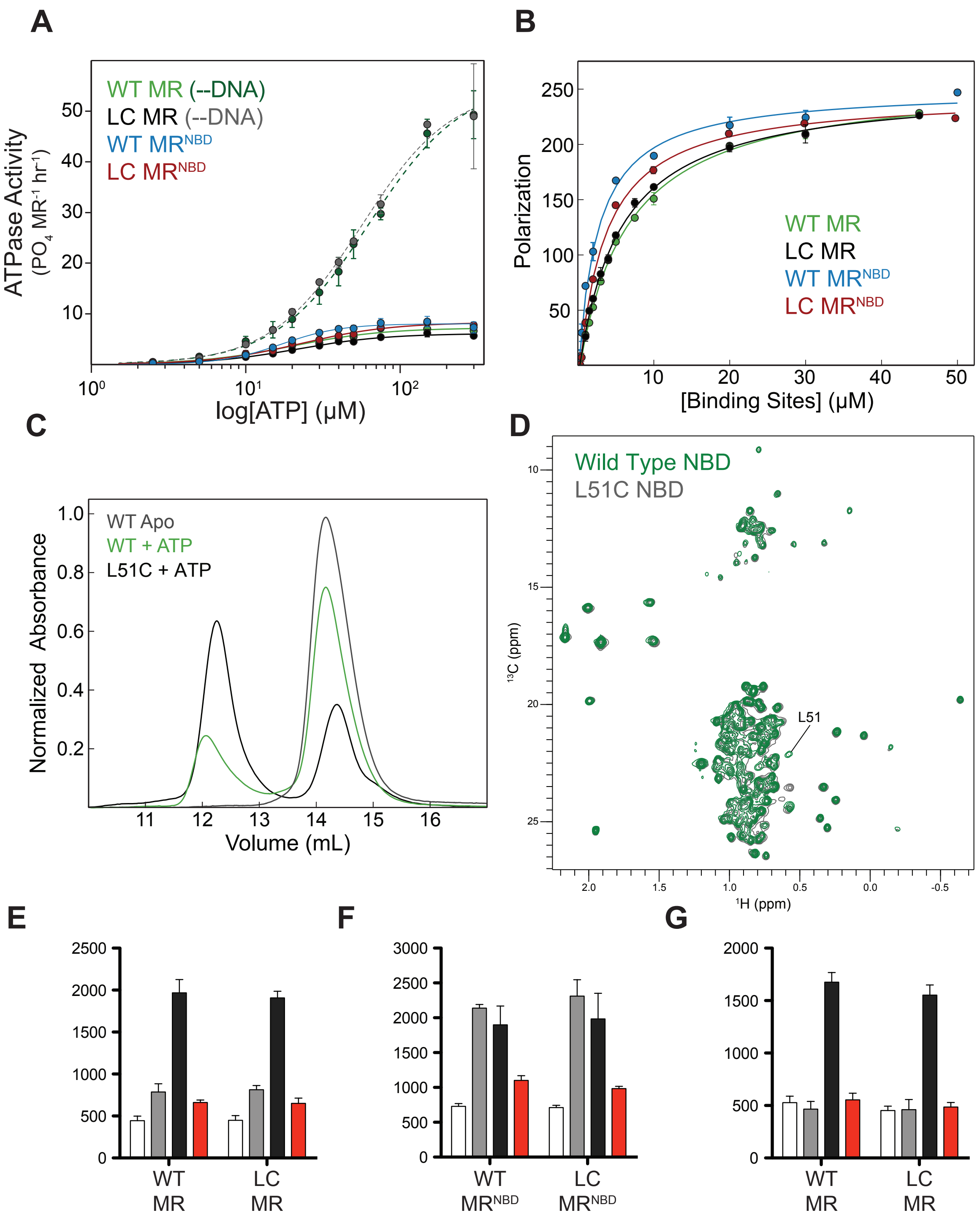

Supplementary Figure 2. The L51C mutation behaves like wild type Rad50. (**A**) Steady-state ATP hydrolysis kinetics of wild type (green) and L51C (black) full-length MR complexes and wild type (blue) and L51C (orange) MR^{NBD} complexes were measured via the colorimetric BIOMOL Green assay. The data points and error bars are the average and standard deviation of three independent measurements, and the solid lines represent fits of the Hill equations to the data (see Methods). Hydrolysis data were collected in the absence (solid lines) and presence

(dashed) lines of linear plasmid DNA. (**B**) Bodipy FL-labeled ATP (5 nM) was titrated with wild type (green) and L51C (black) full-length MR complexes and wild type (blue) and L51C (orange) MR^{NBD} complexes. Fluorescence polarization values (Ex. 485 nm/Em. 530 nm) were fit to the quadratic form of the simple two-state binding equation to obtain binding affinities (solid line). K_Ds of 6.3 ± 0.3 and 5.4 ± 0.4 μM were obtained for full-length wild type and L51C MR, respectively, and 2.6 ± 0.3 and 3.4 ± 0.2 μM were obtained for truncated wild type and L51C MR^{NBD}, respectively. Data points and error bars are the average and standard deviation of three replicates. (**C**) The ATP-dependent dimerization of Rad50 NBDs, in complex with the Mre11 C-terminal helix-loop-helix motif, was observed by size exclusion chromatography. Wild type and L51C chromatograms in the presence of ATP are reported as green and black lines, respectively. Wild type in the absence of ATP is shown as a grey line. (**D**) Overlaid $^{13}C,^{1}H$ HMQC spectra for ILVM-labeled wild type (green) and L51C (gray) $Rad50^{NBD}$ bound to unlabeled Mre11. (**E** and **F**) Full-length MR (**E**) and MR^{NBD} (**F**) exonuclease activity as determined by the Exo2 substrate, where a 2-aminopurine (2-AP) probe is place at the second nucleotide from the 3'-end of a 29 nucleotide double-stranded DNA. Bars denote the resulting 2-AP fluorescence that occurs after cleavage in buffer without Mn^{2+} (white), in the presence of 1 mM Mn^{2+}/5 mM Mg^{2+} (grey), 1 mM Mn^{2+}/1 mM ATP/5 mM Mg^{2+} (black), and 1 mM Mn^{2+}/1 mM AMP-PNP/5 mM Mg^{2+} (red). The bars and errors represent the average and standard deviation of at least three measurements. (**G**) Full-length MR exonuclease activity as determined by the Exo11 substrate, where the 2-AP probe is placed at the 11th nucleotide. The colors of the bars follow (**E** and **F**), and the bars and errors represent the average and standard deviation of at least three measurements.

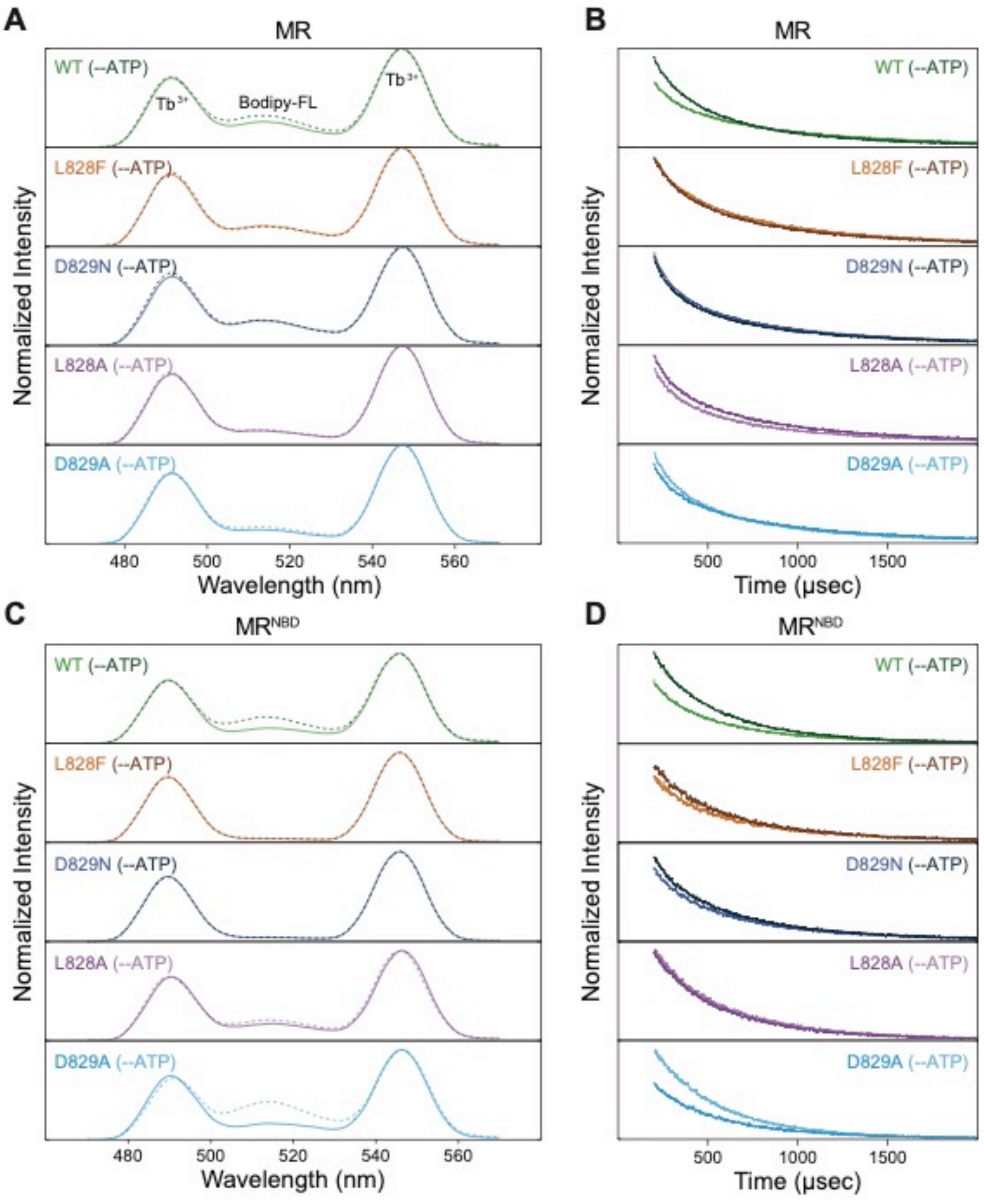

Supplementary Figure 3. LRET and the Rad50 closed state. (**A** and **C**) The emission spectra for wild type (green), L828F (orange), D829N (blue), L828A (purple), and D829A (light blue) Tb^{3+}/Bodipy FL labeled full-length Rad50 (**A**) or Rad50^{NBD} (**C**) in complex with Mre11. Following excitation at 337 nm, emission spectra were recorded from 470 – 570 nm in absence (light color) or presence of 2 mM ATP (dark color). Spectra were normalized to the Tb^{3+} emission peak at 549 nm, and fluorescence at ~515 nm corresponds to donor-sensitized Bodipy FL signal. (**B** and **D**) To determine LRET lifetimes, the donor-sensitized Bodipy FL emission

intensity (520 ± 10 nm) was recorded over time in the absence and presence of 2 mM ATP (light and dark curves, respectively) for full-length MR (**B**) and MR^{NBD} (**D**).

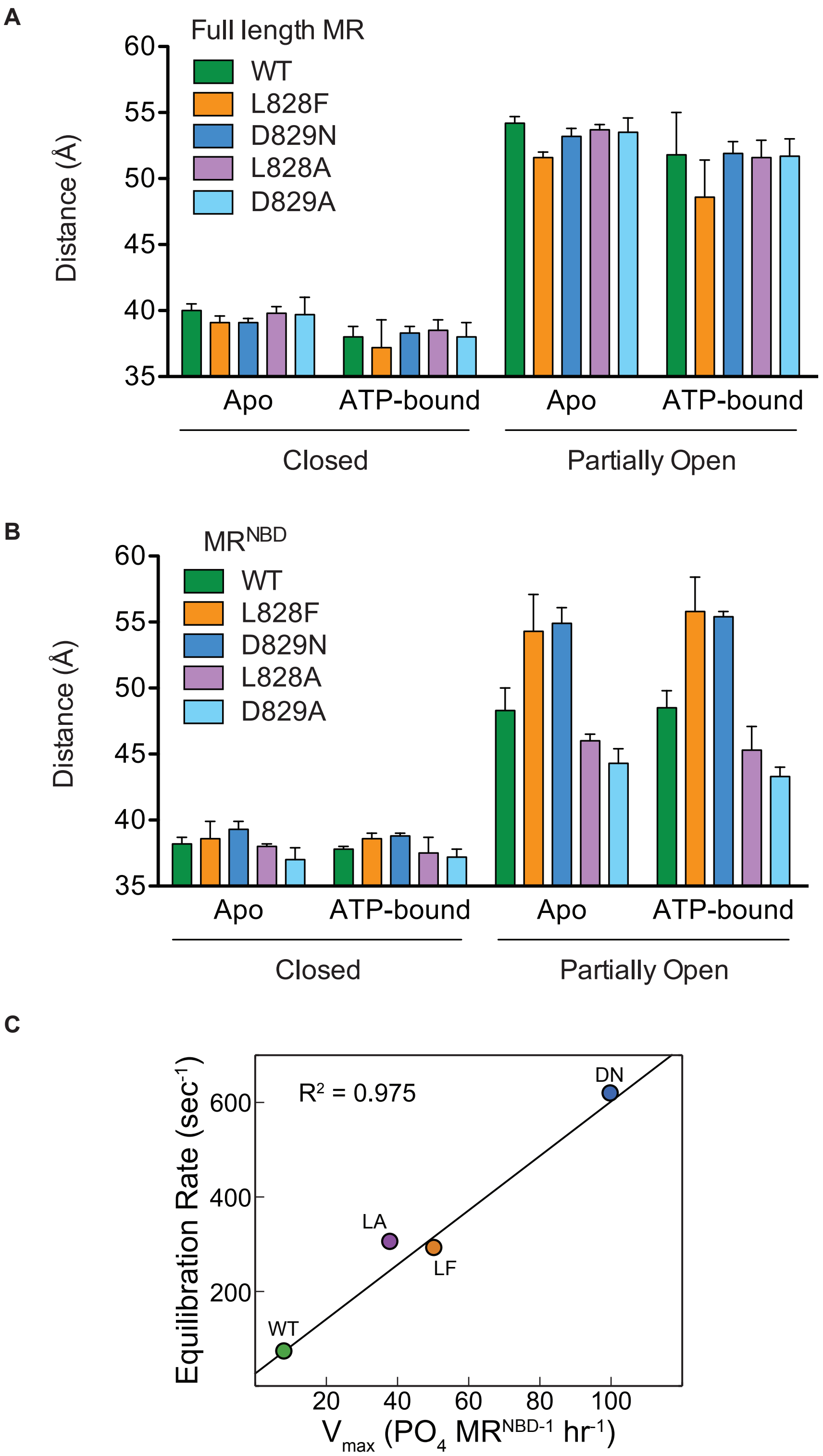

Supplementary Figure 4. The closed state of Rad50. (**A** and **B**) Bar charts of the average distribution for the closed (~38 Å) and 'partially open' conformations (~48 – 55 Å) for full-length MR (**A**) and MR^{NBD} (**B**), which were calculated from the multi-exponential fits listed in Table 1. Errors are the standard deviation of three replicates. (**C**) Scatter plot of the MR^{NBD} ATP-induced

association-dissociation equilibrium rate (Figure 3C) *vs* the MR^{NBD} maximum velocity of ATP hydrolysis (from Figures 2D and 2E). The line represents a linear regression through the points, and the squared Pearson's correlation coefficient is given in the upper left-hand corner.

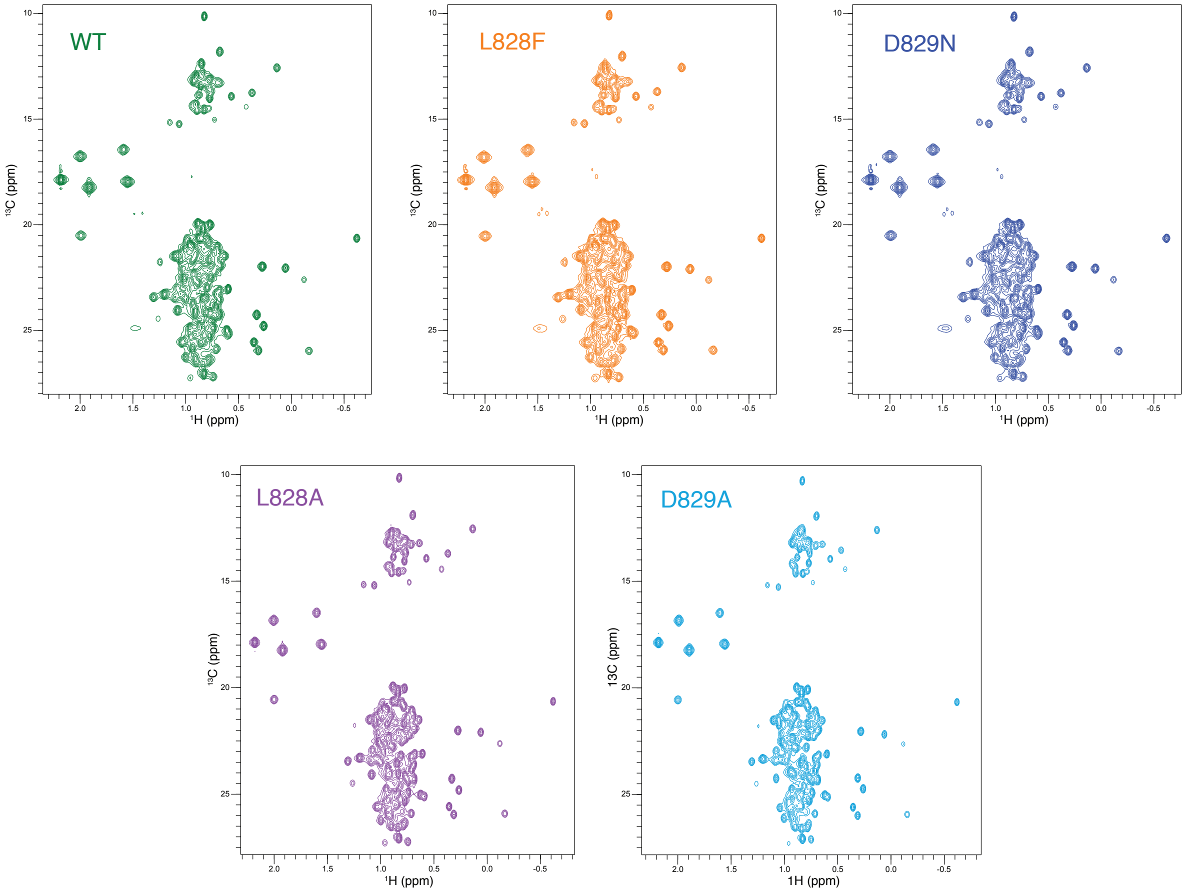

Supplementary Figure 5. ILVM correlation spectra. 2D ^{1}H,^{13}C methyl-TROSY HMQC spectra are shown for U-$^{2}H^{12}C$, Ileδ1-$^{13}CH_3$, Leuδ/Valγ-[$^{12}CD_3$/$^{13}CH_3$], Metε-$^{13}CH_3$ (ILVM)-labeled wild type (green), L828F (orange), D829N (blue), L828A (purple), and D829A (light blue) Rad50^{NBD} in complex with unlabeled Mre11.

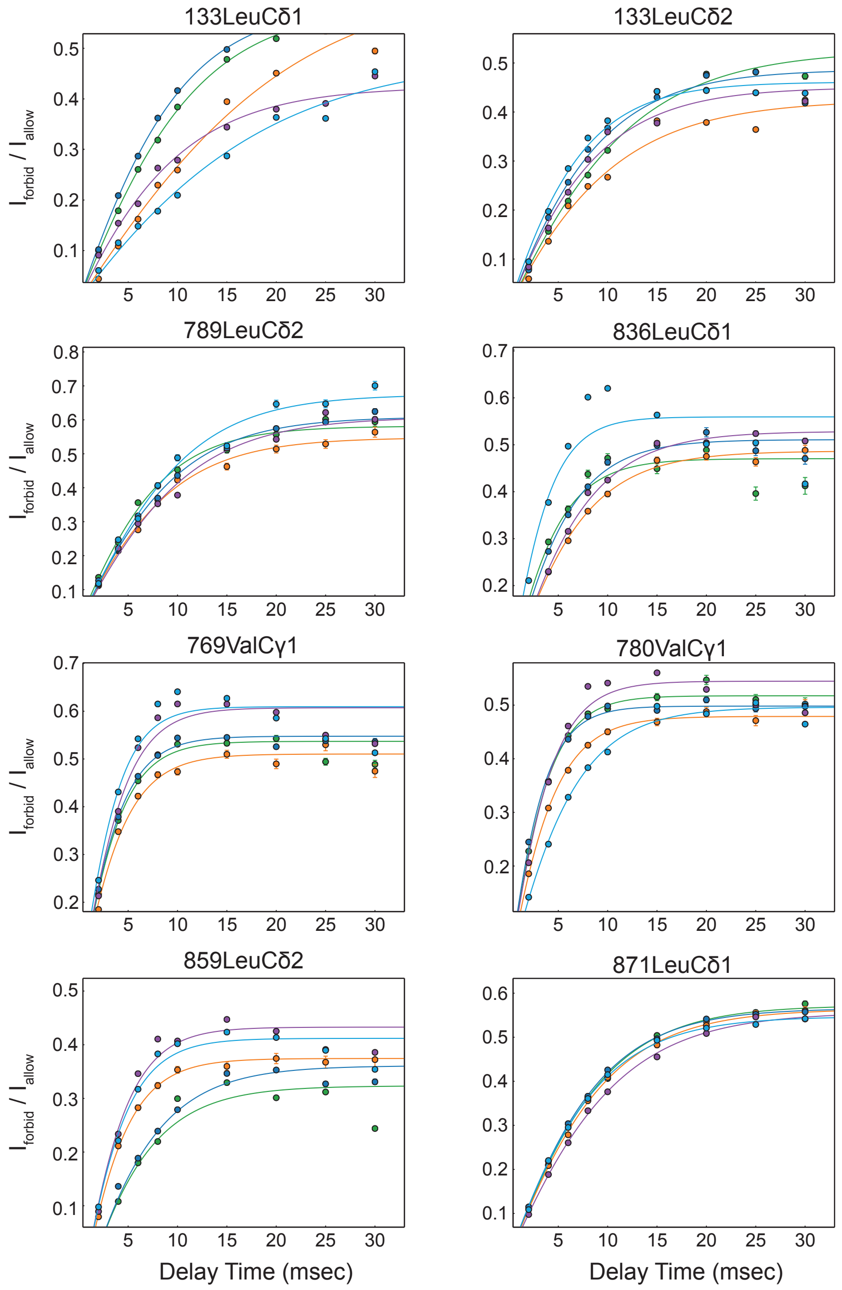

Supplementary Figure 6. Representative relaxation violated, 'forbidden' build up curves. Overlays of methyl ^{1}H 'forbidden' triple quantum transition cross-correlated relaxation build up curve are shown for several $^{13}CH_3$ methyl groups in wild type (green), L828F (orange), D829N (blue), L828A (purple), and D829A (light blue) MR^{NBD} complexes. Errors in each point are derived from the noise in the spectra. Solid lines represent the fits to the data used to calculate η rates.

Supplementary Table 1. Rad50 ATP hydrolysis

A. Full-length *Pf* MR ATP hydrolysis

	Wild Type	L828F	D829N	L828A	D829A
**V_{max}[a]**	7.23 ± 0.35	15.6 ± 0.5	21.4 ± 0.2	11.1 ± 0.3	27.4 ± 0.3
**K_M[b]**	19.8 ± 2.1	49.2 ± 3.5	43.9 ± 0.9	36.7 ± 2.1	39.0 ± 0.7
**n[c]**	1.44 ± 0.20	1.26 ± 0.07	1.42 ± 0.03	1.6 ± 0.12	2.02 ± 0.06
**k_{cat}[d]**	0.12 ± 0.01	0.26 ± 0.01	0.36 ± 0.01	0.18 ± 0.01	0.46 ± 0.01
**k_{cat}/K_M[e]**	6.08 ± 0.72	5.29 ± 0.42	8.11 ± 0.18	5.02 ± 0.33	11.7 ± 0.2
**Turnover[f]**	8.30 ± 0.40	3.84 ± 0.13	2.81 ± 0.03	5.43 ± 0.16	2.19 ± 0.02
**$K_{D,\ ATP}$[g]**	6.3 ± 0.3	2.6 ± 0.1	6.0 ± 0.3	4.0 ± 0.1	5.8 ± 0.3

B. *Pf* MR^{NBD} ATP hydrolysis

	Wild Type	L828F	D829N	L828A	D829A
**V_{max}[a]**	8.06 ± 0.22	50.2 ± 1.2	99.8 ± 1.3	37.8 ± 1.2	67.7 ± 1.4
**K_M[b]**	17.0 ± 0.9	47.1 ± 2.2	44.6 ± 1.2	53.2 ± 3.4	24.5 ± 1.0
**n[c]**	2.24 ± 0.25	1.53 ± 0.08	1.48 ± 0.04	1.35 ± 0.07	1.81 ± 0.11
**k_{cat}[d]**	0.13 ± 0.01	0.84 ± 0.02	1.66 ± 0.02	0.63 ± 0.02	1.13 ± 0.02
**k_{cat}/K_M[e]**	7.90 ± 0.48	17.8 ± 0.9	37.3 ± 1.11	11.8 ± 0.8	46.0 ± 2.0
**Turnover[f]**	7.44 ± 0.20	1.20 ± 0.03	0.60 ± 0.01	1.59 ± 0.05	0.89 ± 0.02
**$K_{D,\ ATP}$[g]**	2.6 ± 0.3	1.3 ± 0.1	3.3 ± 0.4	2.5 ± 0.2	3.4 ± 0.3

C. Full-length *Pf* MR + DNA ATP hydrolysis

	Wild Type	L828F	D829N	L828A	D829A
**V_{max}[a]**	56.0 ± 2.2	38.3 ± 3.0	38.4 ± 3.7	13.8 ± 0.8	54.3 ± 0.7
**K_M[b]**	63.6 ± 4.9	138 ± 25	99.6 ± 21.5	56.7 ± 7.1	46.6 ± 1.2
**n[c]**	1.43 ± 0.09	0.96 ± 0.06	1.06 ± 0.10	1.33 ± 0.13	1.58 ± 0.05
**k_{cat}[d]**	0.93 ± 0.04	0.64 ± 0.05	0.64 ± 0.06	0.23 ± 0.01	0.91 ± 0.01
**k_{cat}/K_M[e]**	14.7 ± 1.3	4.60 ± 0.91	6.43 ± 1.52	4.06 ± 0.56	19.4 ± 0.6
**Turnover[f]**	1.07 ± 0.04	1.57 ± 0.12	1.56 ± 0.15	4.35 ± 0.26	1.10 ± 0.02

D. *Pf* MR^{NBD} + DNA ATP hydrolysis

	Wild Type	L828F	D829N	L828A	D829A
**V_{max}[a]**	15.5 ± 0.2	54.5 ± 0.8	129 ± 2	71.9 ± 3.0	107 ± 6
**K_M[b]**	33.3 ± 0.9	48.8 ± 1.5	66.4 ± 1.8	100 ± 10	30.3 ± 4.0
**n[c]**	1.70 ± 0.06	1.48 ± 0.04	1.38 ± 0.03	0.98 ± 0.04	1.11 ± 0.12
**k_{cat}[d]**	0.26 ± 0.01	0.91 ± 0.01	2.15 ± 0.03	1.20 ± 0.05	1.79 ± 0.10
**k_{cat}/K_M[e]**	7.76 ± 0.23	18.6 ± 0.6	32.4 ± 1.0	11.9 ± 1.3	59.0 ± 8.4
**Turnover[f]**	3.88 ± 0.06	1.10 ± 0.02	0.46 ± 0.01	0.83 ± 0.03	0.56 ± 0.03

[a] μM PO_4 h^{-1} μM MR^{-1}, [b]μM, [c]Hill coefficient, [d]min^{-1}, [e]min^{-1} μM^{-1} x 10^{-3}, [f]min, [g]μM. The reported values and their errors represent the average and standard deviation of at least three measurements.
